# Fractionated proteomics identifies a protein network mitigating resistance exercise-induced damage in human skeletal muscle

**DOI:** 10.1038/s41467-026-75501-y

**Published:** 2026-07-28

**Authors:** Maithreyan Kuppusamy, Daniel Jacko, Yamini Gupta, Sandro Sieger, Kirill Schaaf, Martin Matijass, Käthe Bersiner, Jonas Zacher, Sara Bonini, Miguel Cosenza-Contreras, Peter van der Ven, Wilhelm Bloch, Dieter O. Fürst, Dominic Winter, Jörg Höhfeld, Pitter F. Huesgen, Sebastian Gehlert

**Affiliations:** 1 Central Institute for Engineering, Electronics and Analytics, ZEA-3, Forschungszentrum, Jülich, Germany; 2 Research Center One Health Ruhr, University Alliance Ruhr & University Hospital Essen Medical Faculty, University Duisburg-Essen, Essen, Germany; 3 Department of Molecular and Cellular Sports Medicine, Institute of Cardiovascular Research and Sports Medicine, German Sport University Cologne, Cologne, Germany; 4 Olympic Base Centre, North Rhine-Westphalia/Rhineland, Cologne, Germany; 5 Institute for Cell Biology, Rheinische Friedrich-Wilhelms University Bonn, Bonn, Germany; 6 Department for the Biosciences of Sports, Institute of Sports Science, University of Hildesheim, Hildesheim, Germany; 7 Department of Preventative and Rehabilitative Sports and Performance Medicine, Institute of Cardiology and Sports Medicine, German Sports University Cologne, Cologne, Germany; 8 Faculty of Biology, University of Freiburg, Freiburg, Germany; 9 CIBSS- Centre for Integrative Biological Signaling Studies, University of Freiburg, Freiburg, Germany; 10 Present Address: Department for Chemistry, Institute of Biochemistry, University of Cologne, Cologne, Germany

**Keywords:** Chaperone-mediated autophagy, Protein-protein interaction networks, Intermediate filaments, Muscle, Diagnostic markers

## Abstract

Resistance exercise (RE) improves strength and muscle mass, with multiple benefits for human health. However, intense RE also induces acute myofibrillar damage. The molecular mechanisms that preserve, mark, degrade, and restore damaged proteins to keep skeletal muscle working under RE are incompletely understood. Based on repeated sampling of human skeletal muscle, we show here that acute, repeated and interrupted RE induce dynamic changes of the protein landscape associated with the sarcomeric cytoskeleton. These changes correlate with changes in phosphorylation indicative of adaptation and deadaptation signaling footprints. Regulation mainly affects the protein network linked to the muscle maintenance protein BAG3, which includes mechanosensory proteins, small heat shock proteins, and a lipid droplet associated protein. All network components exhibit altered phosphorylation and increased cytoskeletal association after damaging RE. Moreover, network components cooperate to recognize strained skeletal muscle structures and mediate their degradation through chaperone-assisted selective autophagy (CASA). Our study thus identifies key regulators of skeletal muscle homeostasis in humans.

## Abstract (web summary)

This study reveals molecular responses of untrained, adapted and deadapted human skeletal muscle to resistance exercise, which identifies a network of mechanosensory proteins and molecular chaperones that ensures skeletal muscle homeostasis.

## Introduction

Resistance exercise (RE) provides a unique stimulation pattern on skeletal muscle that acutely augments myofibrillar protein synthesis and, in the long term, increases muscle fiber size and strength. RE offers multiple benefits for health and well-being, which have led the World Health Organization to strongly advocate the incorporation of RE into daily lifestyle routines across nearly all age groups^1^. Although propagated as “exercise medicine”^2^, intense mechanical stimulation can overload sarcomeres, the smallest contractile units in skeletal muscle, and can result in sarcomere damage^3^. This damage is manifested in ultrastructural changes, including a loss of the regular striated pattern of actin-anchoring complexes at the sarcomeric Z-disc^4^. The sarcomeric Z-disc is a complex mechanosensitive protein network that separates adjacent sarcomeres and fulfils important roles in anchoring force-generating filaments as well as sensing and transducing mechanical forces towards and from the extracellular matrix^5^. Filamin C (FLNC), a highly mechanosensitive actin-binding protein in the Z-disc can further accumulate together with Xin actin-binding repeat-containing proteins (XIRPs: Xin/XIRP1 and XIRP2)^6^ in specific regions of the strained skeletal muscle, also referred to as “lesions”^7–9^. However, which additional proteostatic proteins are associated with lesions in human skeletal muscle, and what mechanistic roles they play, remain to be investigated.

In healthy skeletal muscle, proteostatic mechanisms ensure the efficient stabilization, degradation, repair, and replacement of damaged sarcomeric proteins^10^. Among them, chaperone-assisted selective autophagy (CASA) has emerged as a pivotal mechanism for maintaining muscle under mechanical stress^11^. Mechanically unfolded FLNC is recognized by a chaperone complex composed of HSPA8 (Hsc70), the small heat shock protein (sHSP) HSPB8 (HSP22), and BCL2-associated athanogene 3 (BAG3)^11^. The HSPA8-associated E3 ligase STUB1 (CHIP) then ubiquitinates FLNC, which triggers the recruitment of the autophagy receptor sequestosome 1 (SQSTM1). BAG3 further interacts with synaptopodin-2/myopodin (SYNPO2), which promotes phagophore formation and engulfment of the BAG3-containing chaperone complex, culminating in autophagic degradation^12^. Notably, mutations in both FLNC and CASA components such as BAG3 can result in myofibrillar myopathies, highlighting the importance of this mechanism^13^. We have shown that a single acute bout of intense eccentric RE, imposing high mechanical strain, induced CASA in human muscle within 24 h, whereas low-intensity RE was without effect^4^. In the same study, progressively increased RE over weeks, providing a recurring stimulus, resulted in elevated expression of CASA components, suggesting that the CASA machinery contributes to muscle resistance against recurring mechanical stress.

Short-term adaptation of skeletal muscle to exercise is predominantly post-transcriptionally and post-translationally regulated^14,15^. Long-term RE results in only minimal changes in proteome composition in a human cohort, whereas dynamic processes such as phosphorylation or changes in localization may drive both acute responses to exercise stimuli and long-term adaptation^16,17^. Indeed, the phosphoproteome of human skeletal muscle is highly responsive to exercise. E.g., endurance exercise triggers widespread changes in phosphosignaling^18^, potentiates insulin signaling^19^, and shows common but also distinct responses to sprint and resistance exercises^20^. Importantly, RE-induced phosphosignaling also directly regulates the proteostatic machinery. BAG3 is dephosphorylated in response to acute and unaccustomed RE in human skeletal muscle, an event that is significantly reduced in the trained state^21^. Furthermore, the small heat shock protein HSPB5 (also known as CRYAB), which prevents aggregation and promotes the refolding of proteins such as actin and desmin^22^, is rapidly phosphorylated at Ser59 after acute RE, with the amplitude depending on the intensity and number of muscle contractions^23^. Phosphorylation in the N-terminal domain destabilizes intersubunit interactions in the HSPB5 homo-oligomer, promoting disassembly and thereby increasing surface exposure, which correlates with higher chaperone activity^24^. However, phosphorylation at Ser59 substantially diminishes within 3 weeks of repeated RE as muscles adapt to repeated RE^25^. Of note, HSPB5 was observed to be washed out of skeletal muscle cryosections from samples taken at rest but not after acute RE^23^, suggesting that the RE-induced mechanical strain alters the binding affinity of HSPB5 to client proteins in the inter- or intrasarcomeric cytoskeleton and/or contractile apparatus. We therefore reasoned that identification of additional proteins with similar behavior, i.e., force-dependent phosphorylation and increased binding to the sarcomeric cytoskeleton, could reveal new components contributing to muscle proteostasis. Because acute RE-induced mechanical stress likely alters the binding of chaperones and CASA components towards damaged or unfolded proteins of the sarcomere, we assumed their rapid redistribution towards the sarcomeric cytoskeleton after each RE bout. Consequently, we fractionated the muscle samples into a soluble and sarcoplasmic/cytoskeletal fraction and analyzed RE-dependent changes in localization of those proteins as well as the overall proteome and phosphoproteome in both fractions.

To date, no proteomic study has investigated how adaptation to repeated RE alters the sarcomeric environment at the molecular level, and how persistent these protective adaptations are when RE is paused. This is important because interrupted training phases better reflect the nature of actual human physical activity, even though RE regimens are recommended to be carried out consistently. Here, we analyze human skeletal muscle biopsies obtained before and after a single bout of high-intensity resistance exercise (RE) in the untrained state, following 6 weeks of repeated RE training, and after 3 weeks of detraining. Our data highlight that moderate training effectively prevents myofibrillar damage and blunts the strong phospho-responses induced by unaccustomed overload RE. Unbiased proteomic and phosphoproteomic profiling of fractionated protein extracts further identifies specific proteins, including the small heat shock proteins HSPB1 and HSPB5, the muscle damage marker XIRP1, the PDZ and LIM domain protein 3 (PDLIM3), and Perilipin-5 (PLIN5), to be phosphorylated and associated with the sarcomere after overload RE. Functional studies reveal a critical role of these proteins in regulating autophagy-mediated muscle proteostasis.

## Results

### High-intensity RE results in lesion formation in healthy human skeletal muscle

**Fig. 1:**
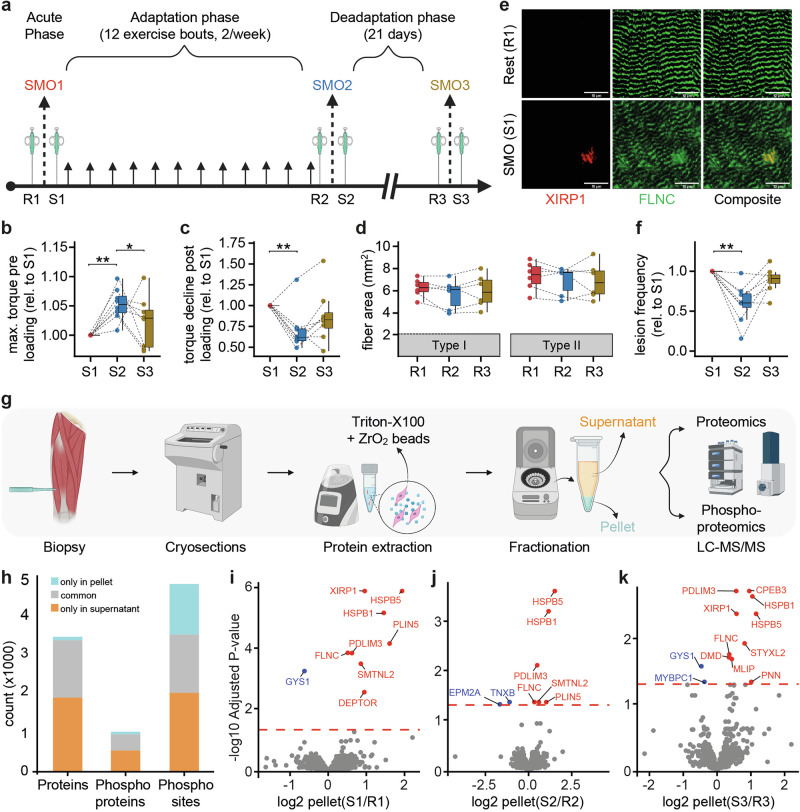
Overview of the study design, effects of RE, and proteomics workflow. **a** Resistance training regime. Biopsies were taken at rest (R), four days before and 60 min after each SMO (S1, S2, S3). The adaptation phase lasted six weeks, with two bouts of RE every week. The deadaptation phase lasted for 21 days. SMO: Standardized Mechanical Overload. Created in BioRender. Kuppusamy, M. (2026) https://BioRender.com/…. **b** Pre-loading torque and **c** fatigue measured before and after every SMO, for each subject normalized to SMO1. **d** Hypertrophy (muscle growth) is measured by the increase in the area of a specific fibre type. **e** Immunolocalization of FLNC and XIRP1 in biopsies taken after SMO (S1). Focal myofibrillar disruptions “lesions” are highlighted by colocalization of XIRP1 and FLNC. The scale bar indicates a length of 10 μm. **f** Percentage of fibres containing lesions after SMO, normalized to the abundance of lesions at S1. **b**–**d**, **f** * denotes individual *p*-value ≤ 0.05, ^**^p-value ≤ 0.01 in an FDR-controlled Friedman test. False discovery rates were controlled using the Benjamini–Krieger–Yekutieli two-stage linear step-up procedure. Exact *p*-Values are provided in the source file. **b**–**d**, **f** Analyses were conducted in *n* = 8 human subjects. **g** Overview of proteome fractionation of muscle biopsies for proteomics and phosphoproteomics using Triton X-100 buffer. Created in BioRender. Kuppusamy, M. (2026) https://BioRender.com/…. **h** Number of proteins, phosphosites, and phosphorylated proteins found in the pellet and supernatant fractions in the proteome and phosphoproteome analyses. Volcano plots show abundance change in the Triton X-100 insoluble cytoskeletal fraction (Pellet) after SMO (S) compared to rest (R) in the **i** untrained state (S1/R1), **j** the adapted state at (S2/R2), and **k** the deadapted state (S3/R3). Proteins with significant accumulation or depletion in response to SMO are highlighted in red and blue, respectively (LIMMA moderated *t*-test <0.05, with FDR < 0.05). **b**–**f** All Box plots illustrate the distribution of data, with the line within each box representing the median. Box plot boundaries define the interquartile range (IQR), encompassing the 25th and 75th percentiles. Whiskers extend to the minimum and maximum data points within 1.5 times the IQR.

We recruited a cohort of eight healthy human participants (seven males and one female) and assessed their individual strengths (participant characteristics are detailed in Supplementary Table 1). Standardized Mechanical Overload (SMO) sessions were performed before and after a 6-week adaptive RE training to study the immediate and long-term regulation of proteostatic regulators after acute SMO-induced stress (Fig. 1a). A final SMO session was conducted after 3 weeks of deadaptation without any RE (Fig. 1a). The adaptive RE training included leg presses, leg extensions, and box jumps. In contrast, the SMO sessions involved twice as many contractions and included single-legged eccentric leg presses, leg extensions, and intense walking down 12 floors (Supplementary Table 2). As expected, the maximal isometric force increased during the adaptation phase and returned to baseline levels during the deadaptation phase (Fig. 1b). Conversely, muscular fatigue levels decreased after adaptation and increased again following deadaptation (Fig. 1c).

Muscle biopsies were collected from the vastus lateralis 4 days before each SMO session (termed “rest” or “R”) and 60 min after each SMO session (termed “post-SMO” or “S”), frozen in isopentane, which was pre-cooled in liquid nitrogen and cryosectioned. Quantification of the average area of type I and II muscle fibers indicated no hypertrophy after adaptation. This was expected, as subjects trained only twice a week for 6 weeks and the training volume of repeated RE was not extensive. We further determined that type I fibers were smaller than type II fibers (Fig. 1d). Importantly, immunostaining for the Z-disc protein FLNC revealed regions with disrupted myofibrils (Fig. 1e), referred to as lesions, following SMO (S1). The well-established muscle damage marker XIRP1^6,8^ specifically accumulates in these lesions after SMO **(**Fig. 1e). The percentage of myofibres with SMO-induced lesions was lower after adaptation (S2) but rebounded after deadaptation (S3) to a level comparable to that after SMO in untrained muscle (S1) (Fig. 1f). Taken together, the physiological and histological data emphasize that SMO training effectively induced the expected myofibrillar damage. Furthermore, repeated RE initiated reversible protective mechanisms mitigating myofibrillar damage, even in the absence of hypertrophic growth.

### Detergent-based fractionation enriches cytoskeleton-associated proteins

To investigate RE-induced changes in protein abundance and phosphorylation state in the context of their subcellular localization, we first homogenized cryosectioned muscle samples in a buffer containing Triton X-100 and fractionated the sample by centrifugation^26^. As expected, this fractionation yields a soluble supernatant proteome fraction and a detergent-resistant pellet fraction enriched in cytoskeleton-associated proteins (Fig. 1g). Both proteome fractions were independently processed and analyzed in data-independent acquisition (DIA) mode, resulting in the identification of 1593 distinct protein groups in the pellet fractions (Supplementary Data 1) and 3436 protein groups in the supernatant fractions (Supplementary Data 2). 98% of the proteins identified in the pellet were also identified in the supernatant fraction (Fig. 1h). From the same samples, phosphopeptides were enriched with titanium dioxide beads and analyzed in data-dependent acquisition (DDA) mode (Fig. 1h). This identified 3583 phosphorylation sites in 970 protein groups in the supernatant and 2854 phosphosites in 493 protein groups in the pellet, again with a large overlap between the fractions (Fig. 1h). Quantitative comparison of proteins found in both fractions validated the efficacy of our fractionation, with proteins enriched in the pellet fraction (Supplementary Fig. 2a) showing functional gene ontology (GO) term categories associated with the sarcomeric cytoskeleton (Supplementary Fig. 2b), while the supernatant fraction was enriched in proteins linked to cytosolic, mitochondrial, and vesicle-related cellular components (Supplementary Fig. 2c). Principal component analysis revealed considerable variability in proteome composition across individual subjects and time points, with a notable distinct clustering of samples from the sole female participant M10 (Supplementary Fig. 2d). The female participant was initially included to maximize sample size, as no prior data suggested sex-specific proteomic responses under the applied conditions. However, we observed that the female participant had only 4% of fibers associated with myofibrillar lesions, which was approximately threefold lower than in the male participants. As this difference likely influenced the regulation of proteostasis factors after SMO in our study, the data from the female participant were excluded from further analyzes.

Given the known variation in proteome composition across different muscle fiber types^27,28^, we assessed MHC isoform abundance at R1 in the proteomics dataset (Supplementary Fig. 1c). Approximately equal proportions of both type I and II fiber types (40–60%) were abundant in all subjects at all time points, except for the predominance of 70–80% fast-twitch fibers observed in subject M1. Fiber type-specific differences in proteostasis factors have been reported during aging and under resting conditions^28^, where BAG3 and HSPB5 abundance is reduced in fast fibers compared to slow fibers. Since our sampling did not allow us to assess the impact of extreme variations in fiber type on the proteome signature in young muscle, we excluded this dataset from further analysis to minimize variability. The proteomics results were corroborated by immunostaining of the myosin heavy chain isoforms MHC I (MYH7) and MHC II (A/X) (MYH1/MYH2) as markers for slow (type I) and fast (type IIA and IIX) fibers, respectively (Supplementary Fig. 1a). The distribution of fiber types varied among study subjects and across time points but did not change significantly over time (Figs. 1d and S1b). We did not differentiate lesion formation between type I and II fibers and whether the mechanism of damage may differ between them. However, the fiber type distribution in the stratified cohort of six subjects was very similar, and we therefore anticipated consistent responses.

### SMO triggers accumulation of selected stress-responsive proteins in detergent-resistant cytoskeletal fractions

Quantitative analysis of the Triton X-100 resistant pellet proteomes before and after SMO revealed eight proteins accumulating in significantly greater abundance (LIMMA-moderated BH adjusted *t*-test *p*-value < 0.05) in untrained muscle (S1/R1), while one protein was less abundant (Fig. 1i). The known muscle damage markers, FLNC and XIRP1, accumulated in response to the first SMO bout (Fig. 1i), consistent with the formation of myofibrillar lesions (Fig. 1e, f). The sHSPs HSPB1 and HSPB5 also accumulated in the pellet. Of note, HSPB1 can substitute for HSPB8 during CASA^21^ and HSPB5, another BAG3-interacting sHSP^29^, was previously observed to associate with the cytoskeleton upon RE^25^. Furthermore, our experiments revealed the SMO-induced accumulation of Smoothelin-like protein 2 (SMTNL2)^30^ and PDZ and LIM domain protein 3 (PDLIM3), two proteins implicated in the organization of actin filaments. The latter protein also has an emerging function in the regulation of actin-dependent membrane trafficking^31^. We additionally observed the accumulation of PLIN5, which is mainly known for regulating lipid droplet homeostasis, mitochondrial cristae density, and fatty acid oxidation^32,33^, and DEPTOR, a repressor of mTOR activity^34^. Notably, the proteins that accumulated in the pellet showed no change or a non-significant reduction in abundance within the soluble fraction (Supplementary Fig. 3). Hence, the increased abundance in the pellet fraction likely arises from enhanced association to cytoskeletal proteins, i.e., altered localization of a fraction of a larger cytosolic pool, rather than de novo synthesis within 60 min. A single protein, glycogen synthase 1 (GYS1), was significantly depleted in the pellet fraction (Fig. 1i), in line with the expected mobilization of glycogen reserves during SMO^35^.

After repeated RE in trained muscle (S2), only six proteins showed an increased association with the pellet fraction in response to SMO (Fig. 1j). These were a subset of the proteins found accumulating in untrained muscle (S1), i.e., FLNC, HSPB1, HSPB5, PDLIM3, PLIN5, and SMTNL2 (Fig. 1j). Consistent with the lower proportion of SMO-induced XIRP1-positive lesions observed in trained muscle (Fig. 1f), XIRP1 did not accumulate significantly under these conditions (Fig. 1j). This further suggests that the accumulating proteins do not exclusively localize to damage-induced lesions but more widely to the mechanically strained sarcomeric cytoskeleton. Two proteins were less abundant in the pellet fraction of trained muscle after SMO (Fig. 1j). These were Tenascin-X (TNXB), which mediates interactions between muscle cells and the extracellular matrix^36^, and Laforin (EPM2A), a glycogen phosphatase that prevents glycogen hyperphosphorylation and supports glycogen breakdown^37^. Because RE dominantly depends on anaerobic glycolysis, Laforin mobilization might reflect the acute involvement of glycolysis during increased energy demand during SMO.

After detraining (S3), SMO re-induced the accumulation of ten proteins in the sarcomeric fraction (Fig. 1k). Along with FLNC, HSPB1, HSPB5, and PDLIM3 (Fig. 1k), XIRP1 was again among the accumulating proteins, consistent with a higher number of lesions at this time point (Fig. 1f). Additionally, we observed a significant accumulation of dystrophin (DMD) and the pseudokinase serine/threonine/tyrosine-interacting-like protein 2 (STYXL2), which is implicated in regulation of sarcomere assembly^38^. Also, the muscle-enriched A-type lamin-interacting protein (MLIP), involved in myoblast differentiation and myonuclear positioning^39^, the cytoplasmic polyadenylation element binding protein 3 (CPEB3), and pinin (PNN), which is a cytoskeletal protein associated with desmosomes^40^ and also part of the exon junction complex regulating DMD splicing^41^, were increased in the pellet. Similar to untrained muscle after SMO1, GYS1 was depleted in the pellet fraction after SMO3, along with the Myosin-binding protein C (MYBPC1), which binds to and stabilizes myosin and actin filaments^42^ (Fig. 1k).

**Fig. 2:**
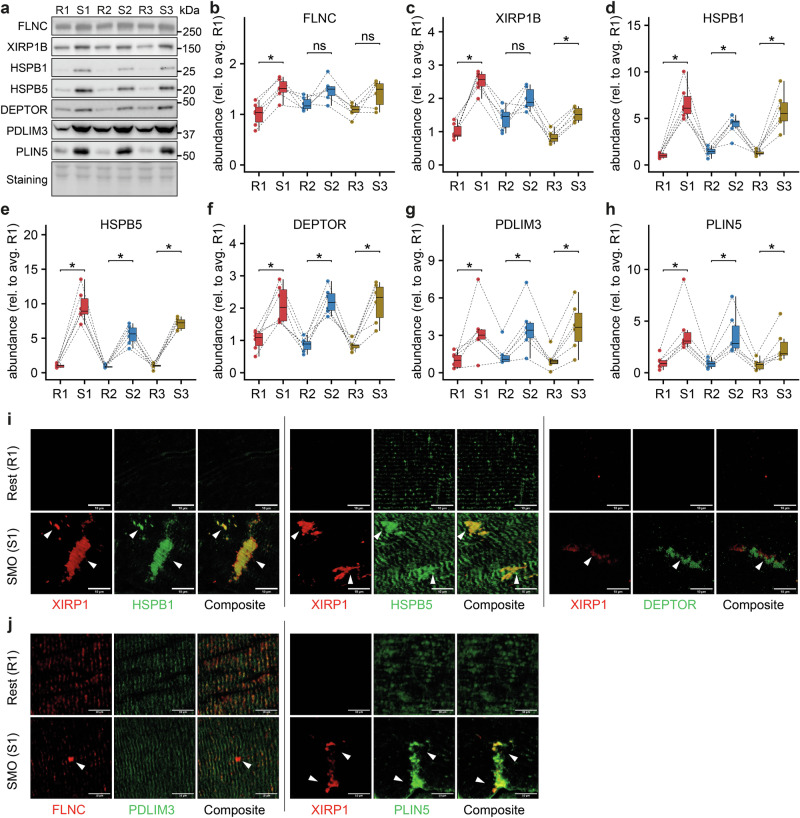
Validation and subcellular location of proteins with increased association to the cytoskeleton. **a** Exemplary immunoblots of FLNC, XIRP1B, HSPB1, HSPB5, DEPTOR, PDLIM3 and PLIN5. Quantitative analysis of immunoblots with antibodies detecting **b** FLNC, **c** XIRP1B (XIRP1A was not detected at this exposition time), **d** HSPB1, **e** HSPB5, **f** DEPTOR, **g** PDLIM3 and **h** PLIN5 immunoblots in the pellet fraction after SMO in the stratified cohort (*n* = 6 independent human subjects). Western blots were performed in technical triplicates. * Indicates two-tailed Wilcoxon matched-pairs signed rank tests *p*-value ≤ 0.05. Immunostaining of muscle biopsies sampled from untrained muscle after SMO (S1) or at rest (R1) for **i** XIRP1 and HSPB1, HSPB5, or DEPTOR, and **j** for FLNC and PDLIM3, or XIRP1 and PLIN5. Overlay images with XIRP1 or FLNC visualize localization to lesions. The scale bar indicates a length of 10 μm. Stainings were performed in technical duplicates in skeletal muscle samples of two independent participants. **b**–**h** All Box plots illustrate the distribution of data, with the line within each box representing the median. Box plot boundaries define the interquartile range (IQR), encompassing the 25th and 75th percentiles. Whiskers extend to the minimum and maximum data points within 1.5 times the IQR.

We further validated these observations by immunoblot analysis of selected accumulating proteins (Fig. 2a). Quantification of FLNC (Fig. 2b), XIRP1B (Fig. 2c, XIRP1A was not reliably detected in the immunoblots), HSPB1 (Fig. 2d), HSPB5 (Fig. 2e), DEPTOR (Fig. 2f), PDLIM3 (Fig. 2g) and PLIN5 (Fig. 2h) showed significant accumulation after SMO1 and often also at the other time points, in agreement with the mass spectrometry results. Consistently, all proteins were reduced in the supernatants (Supplementary Fig. 3). The SMO-induced accumulation of HSPB1 and HSPB5 in the pellet depended on the training state, with reduced accumulation in the pellet after adaptation (S2) (Fig. 2d, e). This agrees with our earlier study, where HSPB5 showed reduced binding to myofibrils after repeated RE^25^.

We subsequently examined the spatial distribution of these proteins within muscle fibers following the initial SMO bout (S1, Fig. 2i, j). Immunostaining showed colocalization of HSPB1 with XIRP1 in myofibrillar lesions (Fig. 2i), similar to the colocalization of FLNC with XIRP1 (Fig. 1e). HSPB5 similarly exhibited pronounced colocalization in lesions but was also observed more dispersed within and across sarcomeres (Fig. 2i). This suggested highly specific SMO-induced relocalization of these chaperones to the pellet fraction, presumably by binding to proteins which were mechanically unfolded upon acute SMO. In contrast, the mTOR inhibitor DEPTOR was distinctly found at the periphery of XIRP1-positive lesions (Fig. 2i). The sequestration of DEPTOR to the lesion margins may contribute to regulating mTOR-dependent tissue repair by temporally and spatially inhibiting mTOR as previously suggested^43^. Consistent with the proteomics data, immunoblot analyzes of the supernatant fractions indicated a significant depletion of FLNC, XIRP1B, HSPB1, HSPB5, DEPTOR, PDLIM3, and PLIN5 after SMO in untrained muscle (Supplementary Fig. 4). In the trained state (after SMO2/S2), only FLNC and XIRP1B showed a significantly decreased abundance in the soluble protein pool. Following detraining (S3), SMO-induced depletion of soluble proteins was again observed for HSPB1, HSPB5, and PLIN5 (Supplementary Fig. 4).

Both HSPB1 and HSPB5 are known interaction partners of BAG3^29^, the central component in the CASA machinery^10^. Since we previously observed a rapid degradation of FLNC, BAG3 and other CASA components in acutely damaged human skeletal muscle fibers^4^, we hypothesized that BAG3 and other CASA components might also be recruited to the pellet fraction. Indeed, immunoblotting showed a significant accumulation of BAG3 and HSPB8 in the pellet fraction, particularly in the untrained (S1) and de-adapted states (S3) (Supplementary Fig. 5a–c), which was mirrored by a tendency to decrease in the soluble fraction (Supplementary Fig. 5a, d, e). Similar changes in BAG3 and HSPB8 abundance are also observed in our proteomics data but did not meet the stringent significance criteria (Supplementary Table 3 and Supplementary Data 4). Immunolocalization studies further corroborated that BAG3 accumulated in lesions, but was also distributed across the entire Z-disc of sarcomeres (Supplementary Fig. 5f). In addition, we observed increased staining for the autophagy receptor SQSTM1 in the vicinity of XIRP1-stained lesions (Supplementary Fig. 5g). Thus, CASA appears to contribute to the maintenance of sarcomere integrity and to lesion remodeling after damaging RE in human muscle also in our training regime.

### Intensity of SMO-induced phosphosignaling depends on the RE training status

**Fig. 3:**
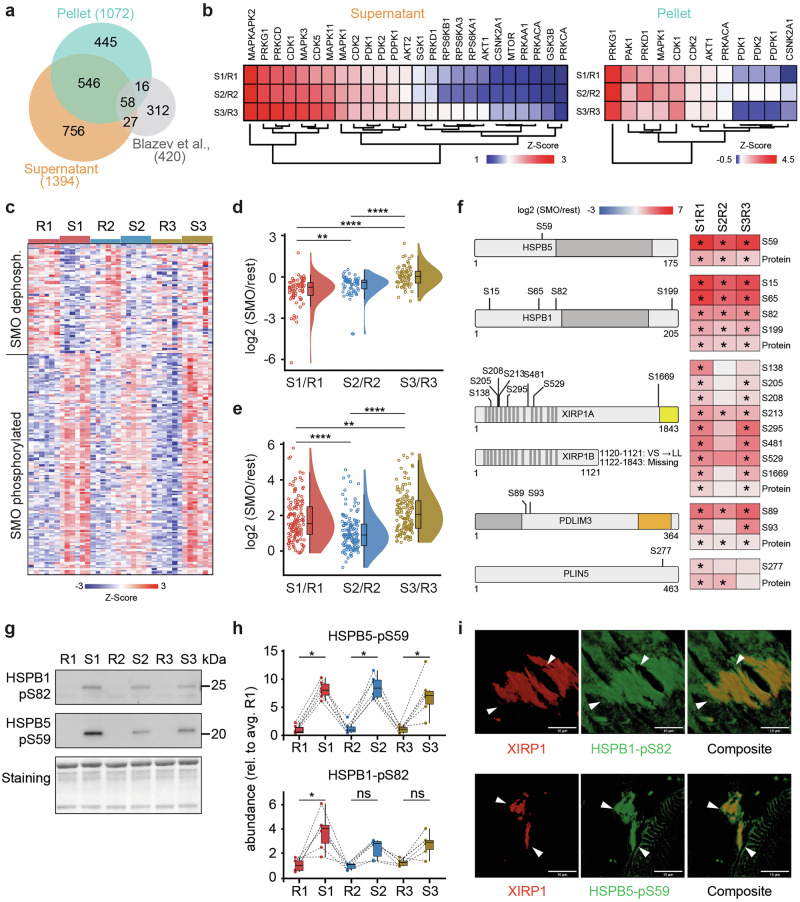
Phosphoproteomics results and kinase activity. **a** Venn diagram showing the number of phosphosites quantified consistently in at least 70% of the replicates in pellet and supernatant, and the number of phosphosites reported as regulated by RE by Blazev et al.^20^. **b** Kinase-Substrate Enrichment Analysis indicating kinases active in the supernatant and pellet after each SMO compared to rest. Positive Z-scores mean higher activity after SMO, and negative Z-scores show lowered activity after SMO. **c** Hierarchical clustering of Z-score normalized intensities of phosphosites significantly changing in the pellet (ANOVA test *p*-value < 0.05 with Tukey’s post-hoc test FDR < 0.05). Raincloud plots show the distribution of regulated phosphosites that are significantly **d** dephosphorylated and **e** phosphorylated in response to SMO in untrained (S1/R1), adapted (S2/R2), and deadapted (S3/R3) states (Pairwise Wilcoxon test, ^**^p < 0.01, ^****^p < 0.0001, ns – non significant). **f** Regulated phosphosites in selected proteins with increased association with the cytoskeleton after SMO. Change in abundance is indicated by colour scale, * marks time points with significant changes in phosphorylation (ANOVA test, followed by Tukey’s post-hoc test with FDR < 0.05) or protein abundance (LIMMA moderated *t*-test <0.05 with FDR < 0.05). Yellow highlights the FLNC-binding domain of XIRP1, orange the LIM domain of PDLIM3. Dark grey boxes indicate the α-crystallin domain in HSPB1 and 5, the PDZ domain in PDLIM3, and grey lines the XIN repeats in XIRP1. Created in BioRender. Kuppusamy, M. (2026) https://BioRender.com/…. **g** Exemplary immunoblots for HSPB5 phosphorylation at S59 (HSPB5-pS59) and HSPB1 phosphorylation at Ser82 (HSPB1-pS82). **h** Quantification of HSPB5-pS59 and HSPB1-pS82 immunoblots in the stratified cohort (*n* = 6 independent human subjects). * Two-tailed Wilcoxon matched-pairs signed rank test *p*-value ≤ 0.05. Box plots illustrate the distribution of data, with the line within each box representing the median. Box plot boundaries define the interquartile range (IQR), encompassing the 25th and 75th percentiles. Whiskers extend to the minimum and maximum data points within 1.5 times the IQR. **i** Immunohistochemical staining for XIRP1 and HSPB5-pS59 and HSPB1-pS82, respectively, after SMO. The scale bar indicates a length of 10 μm. Stainings were performed in technical duplicates in skeletal muscle samples of two independent participants.

Because altered phosphorylation drives changes in protein function and localization following physiological stress, we hypothesized that the adaptive and de-adaptive processes induced by damaging RE in untrained muscle (SMO1), after repeated training (SMO2), and after detraining (SMO3) would be characterized by differential phosphosignaling. To identify such changes, we constrained the soluble and pellet datasets to phosphosites quantified in at least 4 of the 6 subjects studied. These selection criteria restricted the dataset to 1072 phosphosites from 257 protein groups in the pellet fraction (Supplementary Data 3) and 1394 phosphosites from 515 protein groups in the soluble fraction (Supplementary Data 4), with 604 sites common to both datasets (Fig. 3a). Notably, 101 of these phosphosites were previously reported as regulated by low-intensity RE^20^. All quantified phosphopeptides and their respective ratios towards resting conditions (S1/R1, S2/R2, and S3/R3) were investigated by kinase-substrate enrichment analysis (KSEA)^44,45^, which suggested an increased activity of cGMP-dependent protein kinase 1 (PRKG1), protein kinase C delta (PRKCD), MAP kinases, and cyclin-dependent kinases in the supernatant. In contrast, the AMP-activated kinase PRKAA1, the cAMP-dependent kinase PRKACA, the glycogen synthase kinase GSK3B, and protein kinase C alpha (PRKCA) appeared less active (Fig. 3b). Intriguingly, also prominent cellular kinases promoting protein translation and cell growth, including AKT1, mTOR and three isoforms of the ribosomal protein S6 kinase (RPS6KA1, RPS6KA3, RPS6KB1) appeared less active 1 h after acute SMO training (Fig. 3b). The reduced activity of such kinases appears counter-intuitive, but likely strongly depends on the timing of biopsy sampling. Indeed, previous studies observed an acute but transient repression of growth-promoting mTOR signaling early after RE, followed by increased activation at a later time point^20,46^. This suggests that energy-consuming muscle anabolism might transiently shut down during the acute phase after exercise stimulation, a time when structural reorganization is required. KSEA of phosphosites in the pellet fraction largely mirrored the altered kinase activities in the supernatant (Fig. 3b). PRKG1 appeared as the strongest increase in activity, whereas 3-phosphoinositide-dependent protein kinase 1 (PDPK1) and casein kinase II seemed less active. In general, SMO-induced changes in kinase activity in the pellet were reduced after adaptation (S2) but increased again after deadaptation (S3) (Fig. 3b).

Quantitative comparison identified 207 phosphosites in 88 proteins in the pellet (Fig. 3c) and 178 phosphosites in 96 proteins in the supernatant (Supplementary Fig. 6a) with significant changes in phosphorylation state in response to SMO at one or more of the studied time points (ANOVA *p*-value < 0.05 with Tukey’s Post Hoc test FDR < 0.05). Interestingly, the phosphorylation sites targeted by SMO-induced changes remained consistent across different training states, indicating similar phosphosignaling in response to acute resistance exercise (RE). However, the response magnitude was diminished in the pellet during the adapted state (S2/R2) compared to both the untrained (S1/R1) and deadapted states (S3/R3) (Fig. 3d–f). In contrast, changes in phosphosignaling in the soluble fraction were more modest, with similar responses across the conditions (Supplementary Fig. 6b, c). GO-CC term analysis indicated that phosphoregulation-targeted proteins were associated with sarcolemma, myofibrils, and the cytoskeleton in both the pellet (Supplementary Fig. 7a) and the soluble fractions (Supplementary Fig. 7b). This was irrespective of the directionality of change, i.e., increased phosphorylation or dephosphorylation (Supplementary Fig. 7).

### Changes in phosphorylation status correlate with altered cytoskeleton association

Notably, SMO-induced phosphorylation, often affecting multiple sites, was consistently observed in five proteins with increased abundance in the detergent-resistant pellet fractions, i.e., HSPB1, HSPB5, XIRP1, PDLIM3 and PLIN5 (Fig. 3f). Increased HSPB1 phosphorylation was observed at S15, S65, S82, and S199 in both pellet (Fig. 3f) and soluble (Supplementary Fig. 6d) fractions. Phosphorylation at S15 and S82 promotes the dissociation of homo-oligomeric HSPB1^47^ and its recruitment to the cytoskeleton^48^ and increases its binding affinity to unfolded FLNC^49^. Similarly, HSPB5 was phosphorylated at S59, a site known to promote its chaperone activity^24^ and to regulate mechanical stress protection in trained skeletal muscle fibers^23,25^. Immunoblotting with phosphosite-specific antibodies validated our mass spectrometry data (Fig. 3g). Unaccustomed SMO1 (S1) induced a strong accumulation of pS82-HSPB1 in the pellet fraction of untrained subjects but was restrained after adaptation (S2) (Fig. 3h). In the supernatant, the increase was more modest, and a significant amount of pS82-HSPB1 was already observed in the resting state (R) (Supplementary Fig. 6e, f). In contrast, HSPB5 showed a much stronger, about eightfold SMO-induced increase in S59 phosphorylation in both pellet (Fig. 3h) and supernatant (Supplementary Fig. 6g) that was less dependent on training status. In line with these observations, immunostaining showed an increased association of pS82-HSPB1 and pS59-HSPB5 with the cytoskeleton, including a strong colocalization with XIRP1-positive lesions (Fig. 3i). This increased site-specific phosphorylation supports the interpretation that phosphorylated HSPBs associate with unfolded and/or aggregating proteins.

The functional relevance of the observed contraction-sensitive phosphosites of PDLIM3, PLIN5, and the lesion marker XIRP1 is not known. All but two of the sites showed an alleviated response in the pellet after repeated RE training, which was reversed upon 3 weeks without RE (Fig. 3f). This indicates that phosphorylation might facilitate binding to mechanically strained sarcomeres and to sites of myofibrillar damage. Most of these phosphorylation events were not observed in the supernatant or showed only a more moderate response, such as PDLIM3 phosphorylation at S89 and XIRP1 phosphorylation at S481 (Supplementary Fig. 6d). In contrast, phosphorylation of XIRP1 at S757 was found in the supernatant after each SMO bout (Supplementary Fig. 6d) but not observed in the pellet, further indicating that changes in phosphorylation correlate with altered subcellular localization. To further explore the functional roles of these proteins in proteostasis, we then turned to a cell culture-based model, in which we sequentially silenced them and investigated their involvement in CASA.

### RE-responsive proteins are subjected to autophagic degradation in differentiated muscle cells

**Fig. 4:**
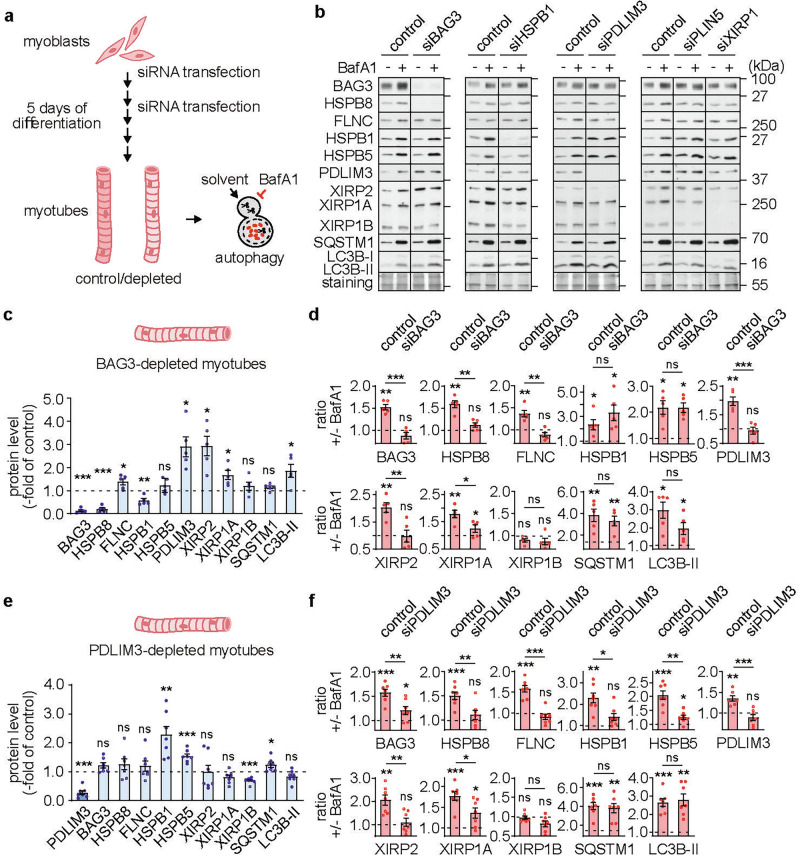
CASA activity in C2C12 myotubes depleted for RE-responsive proteins. **a** Schematic presentation of the transfection and differentiation workflow, and treatment conditions. **b** Exemplary immunoblots for the CASA complex components BAG3 and HSPB8, the CASA substrate FLNC, the RE-responsive proteins HSPB1, HSBP5, PDLIM3, PLIN5, XIRP1A, XIRP1B and XIRP2, the autophagy receptor SQSTM1 and LC3B-I and LC3B-II as autophagy markers. Ponceau S membrane staining is shown as a loading control (staining). Quantification of **c**, **e** protein abundance and **d**, **f** protein turnover as determined by protein abundance with and without BafA1 treatment in **c**, **d** BAG3-depleted and **e**, **f** PDLIM3-depleted myotubes. Protein level in control cells was set to 1 and is represented as a dashed line in **c**, **e**. In **d**, **f** the dashed line represents a value of 1, indicating no change in protein abundance between BafA1-treated and untreated cells. Data are shown as mean values ± SEM, *n* = 5 independent experiments in **c**, **d** and *n* = 7 independent experiments in **e**, **f**. Statistical analysis was carried out using two-tailed unpaired *t*-test with Welch’s correction: ^*^p ≤ 0.05, ^**^p ≤ 0.01, ^***^p ≤ 0.001, ns – non-significant. Asterisks above column bars indicate significance compared to control. Asterisks above column-connecting lines indicate significance between the compared samples.

Given the established role of BAG3-dependent CASA in muscle proteostasis, we speculated that the RE-responsive proteins could functionally interact with the CASA machinery and consequently be subjected to autophagic degradation. To test this, we used murine C2C12 skeletal muscle cells as a cellular model system. When differentiated to myotubes, CASA is activated in these cells due to mechanical forces arising from spontaneous contraction^21^. To assess autophagic turnover, myotubes were treated with Bafilomycin A1 (BafA1), which interferes with the acidification of lysosomes, leading to an inhibition of late stages of autophagy and an accumulation of autophagy clients (Fig. 4a). Immunoblot analysis showed that BafA1 treatment caused an accumulation of FLNC and also lipidated LC3B (LC3B-II), SQSTM1, SYNPO2, HSPB8 and BAG3 (Fig. 4b, d). The latter proteins cooperate during CASA to coordinate the degradation of the CASA client FLNC and are co-degraded during this process^10^. The data demonstrate that the CASA pathway is active in differentiated C2C12 myotubes as previously described^21^. Notably, HSPB1, HSPB5, and PDLIM3 also accumulated after BafA1 treatment, indicating that they are subject to autophagic turnover in differentiated myotubes (Fig. 4b, d).

We further analyzed the expression of XIRPs in myotubes with our XIRP antiserum that recognizes all XIRPs (XIRP1A, XIRP1B and XIRP2). Remarkably, XIRP1A and XIRP2 accumulated in a BafA1-dependent manner (Fig. 4b, d), indicating an autophagy-mediated turnover, possibly through association with the CASA machinery. In contrast, XIRP1B, which lacks a filamin-binding domain, was not significantly affected by BafA1 treatment (Fig. 4b), suggesting an autophagy-independent function distinct from XIRP1A and XIRP2.

### RE-responsive proteins are essential for CASA

We then investigated whether the observed autophagic degradation of HSPB1, HSPB5, PDLIM3, XIRP1A, and XIRP2 was dependent on BAG3 and thus mediated by CASA. BAG3 was efficiently depleted in differentiating myotubes by siRNA transfection (Fig. 4b, c). Consistent with previous results^50^, BAG3 depletion caused a strong decline in the cellular levels of its partner chaperone, HSPB8, reflecting destabilization of the orphaned sHSP (Fig. 4b, c). Moreover, HSPB1 levels were significantly reduced in this setting, suggesting a direct association and functional interplay between BAG3 and HSPB1 in myotubes. In contrast, FLNC, PDLIM3, XIRP2, XIRP1A and LC3B-II all accumulated in BAG3-depleted myotubes (Fig. 4b, c). To monitor autophagic degradation, siRNA-treated myotubes were incubated in the presence of BafA1. As expected, BAG3 depletion abrogated the autophagic turnover of FLNC and HSPB8 (Fig. 4b, d). Yet, the turnover of SQSTM1 and LC3B was not significantly reduced. The core autophagy factors apparently do not require BAG3 for their autophagic turnover, and a basal autophagy pathway is maintained in myotubes deficient for BAG3. Nevertheless, autophagic degradation of PDLIM3, XIRP2, and XIRP1A was significantly attenuated in BAG3-depleted cells (Fig. 4b, d), demonstrating their participation in CASA as clients or executing factors. On the other hand, turnover of HSPB1 and HSPB5 was not reduced upon BAG3 depletion, indicating that these sHSPs can associate with the core autophagy machinery independently of BAG3. This is in line with previous work showing a direct interaction of HSPB1 and SQSTM1^51^, which could circumvent a BAG3 requirement and could also affect HSPB5, known to form hetero-oligomers with HSPB1^52^. Despite a BAG3-independent docking of HSPB1 and HSPB5 onto autophagosomal membranes, the sHSPs may still cooperate with BAG3 during the recognition of cytoskeleton clients. In support of this, the depletion of HSPB1 in myotubes affected the steady-state levels of common network components, including FLNC, HSPB5, PDLIM3, XIRP1A, and XIRP1B (Fig. 4b, and Supplementary Fig. 8a). In addition, HSPB1 depletion, like BAG3 depletion, abrogated the autophagic degradation of FLNC, PDLIM3, and XIRP2 (Fig. 4b, and Supplementary Fig. 8b). These cytoskeletal and cytoskeletal-associated proteins thus require both HSPB1 and BAG3 for autophagic degradation, strongly indicating a functional interplay between HSPB1 and BAG3 during CASA.

It was not possible to verify a critical involvement of HSPB5 in CASA because depletion of HSPB5 during differentiation interfered with myotube formation. However, the observation that HSPB5 is subjected to autophagic turnover in myotubes in a manner dependent on HSPB1 and other network components, as described below (Fig. 4, and Supplementary Fig. 8), strongly suggests that HSPB5 can also actively participate in CASA as a client-recognizing chaperone.

We then tested a possible function of PDLIM3, PLIN5, and XIRP1 during CASA by transient transfection of myotubes with specific siRNAs for these proteins (Fig. 4a, b). Depletion of PDLIM3 significantly affected the steady-state levels of HSPB1, HSPB5, XIRP1B, and SQSTM1, consistent with a central role in the proteostasis network (Fig. 4b, e). Even more strikingly, the autophagic turnover of BAG3, HSPB8, FLNC, HSPB1, HSPB5, XIRP2, and XIRP1A was strongly attenuated upon PDLIM3 depletion, whereas basal SQSTM1- and LC3B-mediated autophagy was unaffected (Fig. 4b, f). PDLIM3 thus emerged as an essential proteostasis factor that acts upstream of the CASA pathway to trigger the degradation of strained cytoskeleton components. This might be directly related to the ability of PDLIM3 to associate with the actin-crosslinking protein α-actinin at Z-discs and to act as a force sensor^53^. Moreover, an interaction of PDLIM3 with the membrane trafficking factor SNX17^31^ could potentially promote membrane recruitment for autophagosome formation during CASA.

Targeting of PLIN5 by siRNA only moderately decreased the cellular pool of the protein by about 20% (Fig. 4b, and Supplementary Fig. 8c). However, this was sufficient to cause an accumulation of HSPB1, HSPB5, and PDLIM3 and decreased levels of both XIRP1 isoforms, demonstrating network involvement (Fig. 4b, and Supplementary Fig. 8c). Remarkably, the depletion of PLIN5, like PDLIM3 depletion, had a global impact on the autophagic degradation of network components, leading to the stabilization of BAG3, HSPB8, FLNC, HSPB1, HSPB5, XIRP2, and XIRP1A (Fig. 4b, and Supplementary Fig. 8d). The findings identify PLIN5 as an integral and essential component of the CASA pathway, demonstrating an unexpected link between lipid droplet biogenesis and CASA. PLIN5 decorates lipid droplets and regulates lipid storage and release^54^. In addition, PLIN5 mediates the interaction between lipid droplets and mitochondria in an exercise-regulated manner to satisfy energy demand^33,55^ and promotes starvation-induced autophagy^56^. Of note, PLIN5 depletion in myotubes inhibited CASA but did not interfere with basal SQSTM1- and LC3B-mediated autophagy, illustrating a specific engagement in autophagosome formation at strained cytoskeleton structures.

Finally, silencing of XIRP1 caused a reduction in the abundance of XIRP1A and XIRP1B by around 75% and affected steady-state levels of other network components, i.e., HSPB1, PDLIM3, and XIRP2 (Fig. 4b, and Supplementary Fig. 8e). Moreover, a global impact on the autophagic degradation of network components was also observed upon XIRP1 depletion (Fig. 4b, and Supplementary Fig. 8f). Turnover of BAG3, HSPB8, HSPB1, HSPB5, PDLIM3, and XIRP2 was significantly reduced in XIRP1-depleted myotubes, and in the case of FLNC, turnover rate was no longer significant following XIRP1 depletion. These findings identified the established muscle damage marker XIRP1 as an essential CASA component.

Several studies have demonstrated that the CASA pathway is activated in response to mechanical forces^4,11,12,21,57–59^. In the murine cell model used here, forces arise from spontaneous myotube contraction, leading to significant CASA activation (Fig. 4, and Supplementary Fig. 8). However, CASA regulation under acute, lesion-inducing mechanical stress has not been analyzed so far. Therefore, C2C12 myotubes were subjected to electrical pulse stimulation under damaging conditions (damage EPS), which has been shown to trigger sarcomeric lesion formation and cytoskeleton damage comparable to RE-induced alterations in human muscle^8^ (see Fig. 2i, j, and Supplementary Fig. 9). Remarkably, CASA activity was strongly attenuated under acute damage EPS (Supplementary Fig. 9b, c). In this situation, autophagic degradation was no longer observed for BAG3, HSPB8, HSPB1, HSPB5 and PDLIM3. An extensive force-dependent co-regulation of the identified BAG3-associated protein network becomes apparent. CASA attenuation under acute and severe mechanical stress may contribute to the accumulation of CASA clients and CASA components in sarcomeric lesions, when rupture prevention is apparently favored over the disposal of damaged proteins. Significant autophagic turnover was only observed for XIRP2 and XIRP1A in this situation (Supplementary Fig. 9b, c). Incorporation of the XIRP proteins in lesions may circumvent the necessity for BAG3-mediated client recognition and sequestration under acute stress and could provide a residual degradation activity, possibly involving a direct crosstalk of XIRPs with the core autophagy machinery. The findings further emphasize the central role of XIRPs in regulating autophagic activity in muscle.

### An extended network of proteostasis factors counteracts mechanically induced damage in human muscle

**Fig. 5:**
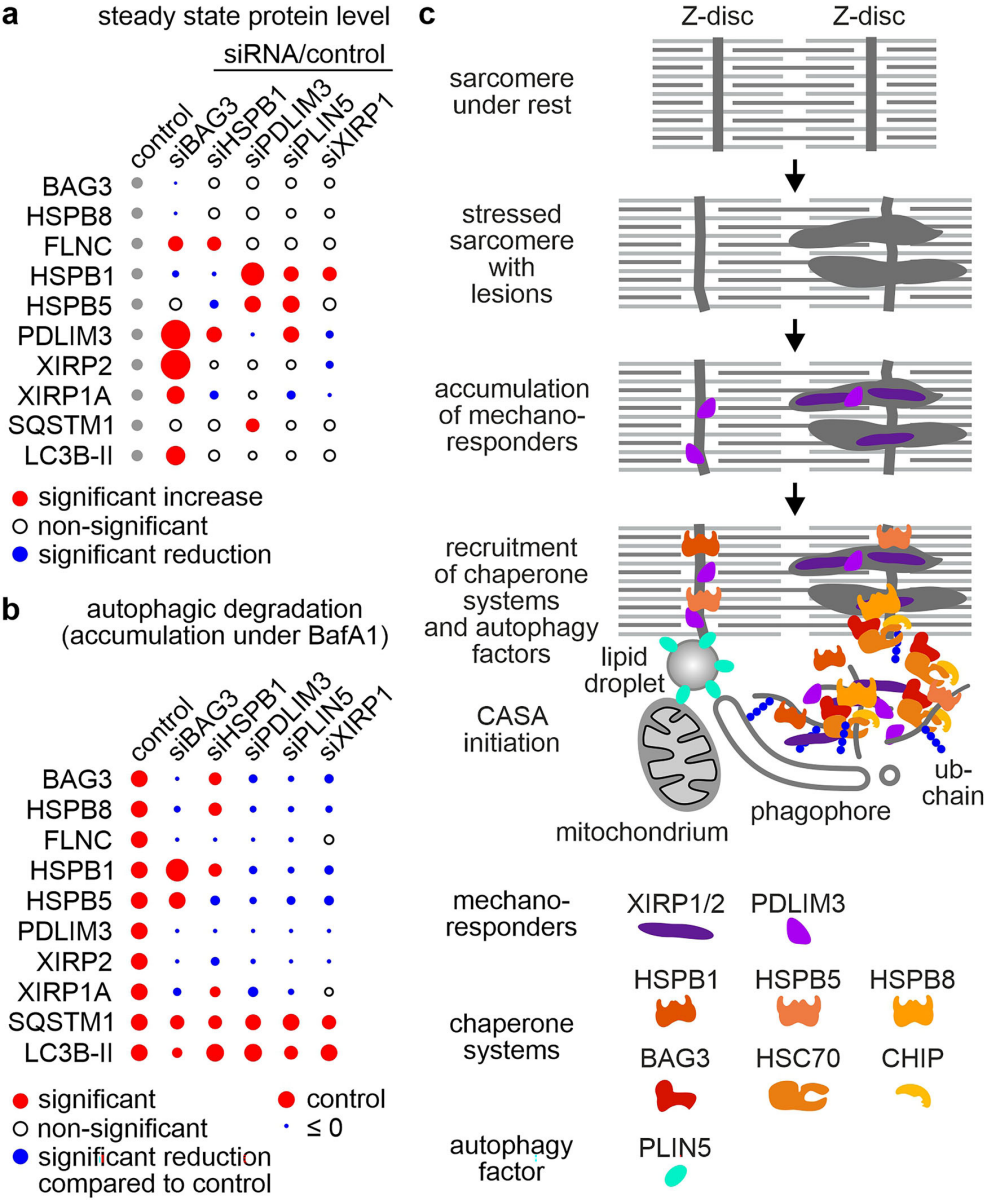
A protein network supporting CASA-mediated degradation. **a** Summary of steady state protein levels in C2C12 myotubes after siRNA-mediated depletion of BAG3, HSPB1, PDLIM3, PLIN5 or XIRP1 as determined in Fig. 4. **b** Summary of autophagic degradation rates in C2C12 myotubes after siRNA-mediated depletion of BAG3, HSPB1, PDLIM3, PLIN5 or XIRP1 as determined in Fig. 4b. **c** Schematic representation of the functional interplay of HSPB1, HSPB5, PDLIM3, PLIN5 and XIRP1 in the CASA-mediated degradation of mechanically damaged muscle proteins (ub-chain – ubiquitin chain).

**Fig. 6:**
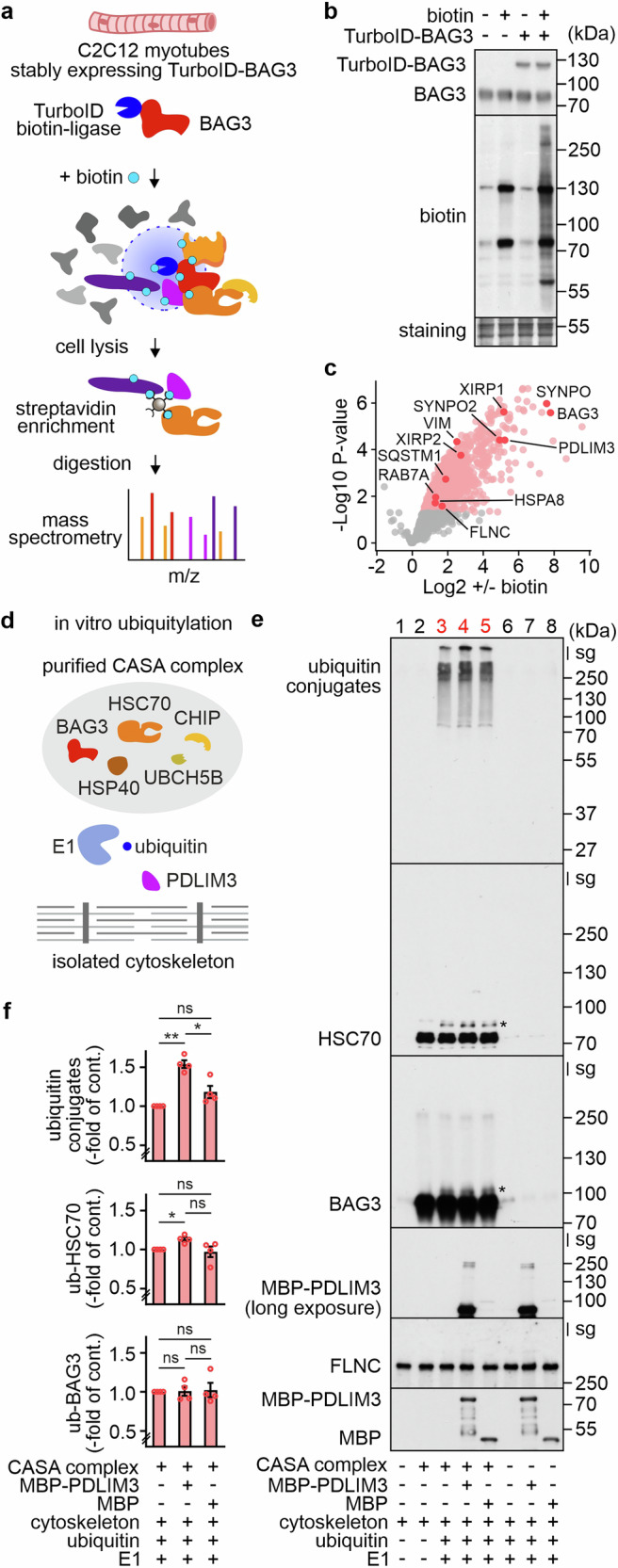
Detection of BAG3-neighboring proteins through proximity biotinylation and functional characterization of PDLIM3 as a stimulating factor in CASA-mediated ubiquitylation. **a** Schematic representation of the proximity biotinylation approach using C2C12 myotubes stably expressing TurboID-BAG3. **b** C2C12 cells, which express TurboID-BAG3 and were treated with biotin as indicated, were analyzed by SDS-PAGE and immunoblotting. Proteins were detected with an anti-BAG3 and anti-biotin antibody. Ponceau S membrane staining is shown as a loading control (staining). Experiments were conducted in triplicates. **c** Volcano plot of streptavidin-purified proteins following proximity biotinylation with TurboID-BAG3. Significantly enriched proteins are shown in light red. Known interaction partners of BAG3 as well as XIRP1, XIRP2 and PDLIM3 are highlighted in red. **d** Schematic representation of the in-vitro ubiquitylation experiments involving purified CASA components, the ubiquitin-activating enzyme E1 and ubiquitin. A cytoskeleton fraction was derived from C2C12 myotubes through differential centrifugation. Impact of PDLIM3 on CASA-mediated ubiquitylation was investigated through addition of purified MBP-PDLIM3 and MBP, respectively. **e** Purified components were combined in ubiquitylation reactions as indicated. Red lane numbers highlight samples that contained the complete ubiquitin conjugation machinery, including E1, the E2 UBCH5B and STUB1 as an E3 ubiquitin ligase. Samples were analyzed by SDS-PAGE and immunoblotting using specific antibodies. Ubiquitin conjugates were detected with an anti-FK2 antibody. Ubiquitylated forms of HSC70 and BAG3 are marked with an asterisk. Ubiquitylated forms of FLNC could not be detected due to the high molecular mass of the protein and the limited separation of proteins of this size range. **f** Quantification of ubiquitin conjugates and ubiquitylated HSC70 (ub-HSC70) and BAG3 (ub-BAG3) as obtained under **e**. Levels in the control sample (lane 3 in **f**) was set to 1. Data are shown as mean values ± SEM, *n* = 4 independent experiments. Statistical analysis was carried out using two-tailed unpaired *t*-test with Welch’s correction: ^*^p ≤ 0.05, ^**^p ≤ 0.01, ns – non-significant. Asterisks above column-connecting lines indicate significance between the compared samples.

Taken together, our proteomic, phosphoproteomic, immunohistochemical, and cell biological data unraveled a network of proteostasis factors that operates in mouse myotubes and human skeletal muscle to restore sarcomere integrity after RE-induced damage (Fig. 5). The network is centered around the CASA pathway, which mediates the degradation of mechanically unfolded and damaged cytoskeleton proteins. Strikingly, our data reveal that CASA activity critically depends on the initial recognition of strained cytoskeleton by PDLIM3 and XIRP1. Both proteins target actin-crosslinking proteins, including α-actinin and FLNC, most likely in a conformation-dependent manner^53^, and their knock-out in mice impairs cardiac muscle homeostasis^60,61^. Under strain, phosphorylated PDLIM3 is increasingly recruited to the actin cytoskeleton, while XIRP1A and XIRP2 accumulate exclusively at sites of myofibrillar damage formed by a strong and localized unfolding of FLNC^8^. Further evidence for the close interplay of BAG3 with XIRPs and PDLIM3 was obtained through proximity biotinylation. C2C12 myotubes that stably express a fusion protein comprising a promiscuous biotin ligase fused to BAG3 (TurboID-BAG3) were cultured in the presence of biotin, resulting in the biotinylation of BAG3-neighboring proteins within a 10–20 nm radius (Fig. 6a). Following purification on streptavidin beads, biotinylated proteins were identified by mass spectrometry. Several known interactors of BAG3 were highly enriched in the biotinylated fraction, including SYNPO, SYNPO2, VIM, SQSTM1, RAB7A, HSPA8/HSC70 and FLNC^11,21,62,63^, validating the approach. Strikingly, XIRP1, XIRP2 and PDLIM3 were also detected as high-scoring BAG3-neighboring proteins (Fig. 6c, and Supplementary Data 5). The mechanoresponsive proteins apparently operate in close proximity to BAG3 in living myotubes. Furthermore, a direct interaction between PDLIM3 and BAG3 was revealed in in-vitro binding experiments (Supplementary Fig. 9d, e). This interaction would facilitate the accumulation of BAG3 at sites of cytoskeletal damage. Subsequent recruitment of HSPB1 and HSPB5 would keep misfolded CASA clients in a state competent for further processing. Multiple interactions of recruited proteins with other CASA components would then facilitate CASA-mediated ubiquitylation and promote contacts with the core autophagy machinery. This would trigger phagophore recruitment and expansion, leading ultimately to the autophagic degradation of damaged muscular structures as a first step towards myofibril repair (Fig. 5). In fact, PDLIM3 stimulated CASA-mediated ubiquitylation in vitro in reconstitution experiments, causing increased ubiquitylation of HSC70 and increased formation of high molecular mass ubiquitin conjugates, most likely representing modified cytoskeleton proteins (Fig. 6d–f). Finally, the association of PLIN5 with sites of cytoskeletal damage could facilitate lipid transfer to nearby mitochondria for energy generation or to phagophore membranes for autophagosome formation.

The close cooperation of the identified network components is also evident in pathological situations. FLNC, XIRP1, BAG3, and HSPB1 are all localized in myofibrillar lesions of a mouse model carrying an FLNC mutation^7^. In addition, XIRP1, XIRP2, HSPB1, and HSPB5 were found together with FLNC and other cytoskeletal proteins as components of pathological protein aggregates in the muscle fibers of myofibrillar myopathy patients^64,65^. BAG3 variants carrying a mutation of P209 accumulate with CASA components in aggresomes in human cells^66^ and in a mouse model of BAG3P209L-induced myofibrillar myopathy^67^, where both XIRP1 and XIRP2 also accumulated massively.

Taken together, while the core of our mechanistic findings derives from mouse myotubes, our study reveals how human skeletal muscle copes with acute stimulation by intense resistance exercise, demonstrates that repeated loading evokes protective adaptations mitigating structural damage, and shows that such adaptations are rapidly reversed upon mechanical unloading.

The human data are observational in nature and limited by cohort size; thus, we cannot exclude potential modulation by fiber type composition, sex, age or other diversity dimensions. Future studies combining fiber type–resolved proteomics or imaging approaches with resistance exercise paradigms will determine whether proteostatic responses differ systematically between fast and slow muscle fibers and sexes. Nevertheless, the concordance between cellular and human data suggests that skeletal muscle adapts to mechanical stress by modulating highly conserved damage-associated processes. Indicative of broader physiological relevance, our proteomic and phosphoproteomic data capture a molecular signature of the well-established “repeated bout effect”— a phenomenon characterized by reduced myofibrillar disruption and soreness upon repeated exposure to initially unfamiliar exercise. In this context, our findings mechanistically complement previous work suggesting that attenuation of muscle damage facilitates long-term hypertrophic remodeling^68^, a central objective not only in athletic but also in clinical settings.

Collectively, our findings expand the concept of skeletal muscle adaptation and deadaptation beyond hypertrophy, providing mechanistic insight into muscle homeostasis at a time when resistance exercise is increasingly advocated for public health.

## Methods

### Subjects

Subjects (24 ± 4.2 years; 184 ± 3.2 cm; 81.5 ± 7 kg; 7 male, 1 female, Supplementary Table S1) were healthy and physically active but refrained from resistance exercise for the lower limbs for at least 4 weeks prior to the study. All subjects identified themselves with their sex and gender written on their individual and signed form of consent. Our study was approved by the ethics committee of the German Sport University of Cologne (005/2018) and was conducted following the guidelines of the Declaration of Helsinki. All subjects provided written informed consent to participate after receiving oral and written explanations regarding the study’s purpose and the potential risks associated with their participation.

### Diet

Subjects fasted overnight and took a standardized meal (Fresubin Energy drink, Fresenius Kabi; 1260 kJ in total, 11.2 g protein, 11.6 g fat, 37.6 g carbohydrates) 1 h before the resistance exercise or three and a half hours before the biopsies, respectively. Drinking water was allowed ad libitum.

### Procedure of the intervention

The intervention comprised three distinct phases (Fig. 1a): an acute exercise phase, an adaptation phase, and a deadaptation phase. During the acute phase, participants experienced a single high-intensity bout (SMO; “Standardized Mechanical Overload”) of resistance exercise (RE) in the untrained state. The following adaptation phase involved RE bouts conducted twice a week, totaling 12 sessions. Finally, participants refrained from RE for 21 days in the deadaptation phase. We conducted single SMOs before the study and at the end of each study phase, followed by skeletal muscle biopsies from the vastus lateralis muscle. The purpose of these SMOs was to deliberately apply a standardized and highly demanding mechanical stimulus to leg skeletal muscle, which induces sarcomeric damage and enables the subsequent analysis of molecular muscle responses under conditions of adaptation and deadaptation. SMO training sessions differed from regular resistance exercise (RE) sessions in that they involved twice the number of contractions, resulting in greater mechanical loading of the skeletal muscle (Supplementary Table S2). The SMOs were scheduled as follows: 1. In the acute phase, as an initial stimulus in the untrained state (SMO1); 2. During early adaptation, after three regular training sessions (this SMO was discontinued in most subjects to minimize the number of biopsies); 3. After completion of the full adaptation phase, i.e., after 12 RE sessions (SMO2); 4. After 21 days of detraining (SMO3). Skeletal muscle biopsies were collected before and after each SMO, providing insights into the molecular signature of the muscle both at rest and in response to acute SMO exposure across different phases of the study. Resting biopsies (R1, R2, and R3) were obtained 4 days prior to SMO1, SMO2, and SMO3, respectively. The corresponding post-loading biopsies (S1, S2, and S3) were collected 1 h after the completion of each SMO session. To assess the functional effects of repeated resistance exercise (rRE), we performed strength testing. Maximal voluntary contraction (MVC) force was measured during isometric leg extension at rest prior to SMO1, SMO2, and SMO3, as well as within the first minute following the completion of each respective exercise session.

### Resistance exercise and Standardized Mechanical Overload

The regular resistance exercise (RE) protocol consisted of four distinct exercises: 1. Leg extensions (Gym80 Signum): one set each at 4, 8, and 16 repetitions maximum (RM); 2. Leg press (MANG Sportgeräte): one set each at 4 and 8 RM; 3. Drop jumps from a 60 cm platform: one set of 10 repetitions; 4. Downstairs walking: descending 182 steps (18 cm each) while skipping every second step; an elevator was used to return to the starting position. Muscle contraction pattern and time under tension in the leg extension and leg press exercises were standardized using a visual biofeedback system. The movement phases were set to 1 s concentric, 2 s eccentric, and 0.5 s for the transition (motion reversal), in order to emphasize skeletal muscle loading. Inter-set rest periods were standardized to 2 min, and inter-exercise rest periods to 3 min. Each session began with a general warm-up on a cycle ergometer (5 min at 1 W/kg body weight), followed by a device-specific warm-up of 10 repetitions at 70% of the individual 10 RM. Training loads were determined 10 days prior to the first SMO and remained constant throughout the intervention, with no progressive overload. All SMO sessions and biopsies were conducted between 8:00 AM and 11:00 AM, whereas regular training sessions were scheduled between 8:00 AM and 6:00 PM.

### Strength testing

Subjects were seated upright in a leg extension device equipped with a force sensor (S-Beam, KM1506 K 5 KKN, Megatron). The lever arm was fixed at a 120° knee angle, and the weight stack was mechanically secured to allow maximal isometric quadriceps contractions. Prior to each trial, subjects positioned their distal shin against the pad of the lever arm. After applying an initial preload of 50 Nm, they were instructed to voluntarily initiate an explosive maximal isometric contraction when ready, maintaining the effort for 3–4 s. To ensure proper stabilization and prevent hip elevation, subjects were allowed to grip the handles located on either side of the seat.

### Human skeletal muscle biopsies

Skeletal muscle biopsies were obtained from the midportion of the vastus lateralis using the percutaneous needle technique^69^. The initial leg selected for biopsy was chosen at random for each subject. Subsequent biopsies were then taken alternately from the contralateral leg. To prevent sampling tissue from previously traumatized biopsy sites during repeated biopsies from the same leg, each new incision was consistently made 2 cm proximal or distal to the prior incision site. Muscle samples were carefully cleaned to remove blood and non-muscle tissues, immediately frozen either directly in liquid nitrogen or in isopentane precooled with liquid nitrogen (for immunolocalization analyzes), and then stored at −80 °C until further processing.

### Protein extraction

The muscle tissue was sectioned using a cryotome at −20 °C. 20 mg of each sample was placed in a pre-chilled 2 mL tube containing 2.8 mm and 1.4 mm zirconium oxide beads (CKMix – 2 mL, P000918-LYSKO-A.0, Bertin Instruments). Each milligram of muscle tissue was homogenized in 30 µL of Triton X-100-based lysis buffer (#9803, CST) supplemented with a protease/phosphatase inhibitor cocktail (#78440, Thermo Scientific) and Phenylmethylsulfonyl fluoride (PMSF). Tissue homogenization was performed using a Precellys 24 homogenizer (Bertin Instruments) with four cycles, each consisting of 15 s at 5800 rpm, interspersed with 120-s cooling intervals on ice. After homogenization, samples were centrifuged at 19,600 × *g* for 15 min at 4 °C to separate the Triton X-100 soluble fraction (supernatant) from the insoluble pellet, primarily containing cytoskeletal muscle components. The supernatant was carefully transferred to a new microcentrifuge tube. The pellet was subsequently washed with 20 µL of Triton X-100 lysis buffer and centrifuged again for 5 min. The resulting second supernatant was combined with the initial supernatant. The pellet was resuspended in a urea buffer (4 M urea, 100 mM Tris-HCl, pH 7.5), supplemented with protease and phosphatase inhibitors (1:100 vol/vol). The volume of urea buffer used corresponded to 70% of the initial Triton X-100 lysis buffer volume. Pellet dissolution was achieved by performing three homogenization cycles, each lasting 10 s at 4500 rpm, with 2-min intervals at room temperature (RT). Protein concentration was measured using the Pierce Bradford protein assay kit (#23225, Thermo Scientific), and samples were stored at −80 °C until further analysis.

### Western blot analysis

For SDS-PAGE (sodium dodecyl sulfate polyacrylamide gel electrophoresis) analysis, protein samples were mixed with 4× Laemmli buffer (#1610747, Bio-Rad) and loaded at 12 µg per well onto Criterion XT 4–12% Bis-Tris gels (#3450125, Bio-Rad), alongside a molecular weight standard (#1610374, Bio-Rad). Electrophoresis was conducted at a constant voltage of 130 V using XT MOPS running buffer (#1610788, Bio-Rad). Proteins were transferred onto PVDF membranes (#10600023, GE Healthcare Life Science) by semi-dry blotting using the Trans-Blot Turbo system (Bio-Rad). Equal protein loading was verified by Ponceau S staining. Membranes were subsequently blocked in 5% nonfat dry milk diluted in Tris-buffered saline (TBS; 150 mM NaCl, 10 mM Tris, pH 7.6) containing 0.1% Tween 20 (TBST). Membranes were incubated overnight at 4 °C with primary antibodies (Supplementary Data 6) prepared in 5% BSA in TBST. After thorough washing with TBST, membranes were incubated for 1 h at room temperature with the appropriate HRP-conjugated secondary antibodies (Supplementary Data 6), diluted in 5% nonfat dry milk in TBST. Following final washes, chemiluminescent detection was performed using an enhanced ECL substrate (#34075, Thermo Fisher Scientific), and signals were captured using a ChemiDoc MP imaging system (Bio-Rad).

### Immunolocalization studies

Frozen muscle tissue was cryosectioned at 7 µm thickness using a cryomicrotome (CM 350 S, Leica Microsystems), and sections were mounted on Polysine™ microscope slides (#631-0107, VWR International). Slides were air-dried for 1 h at room temperature (RT), followed by fixation in pre-chilled acetone (−20 °C) for 8 min. After acetone evaporation, sections were blocked in 5% BSA in TBS for 1 h at RT. Following a TBS wash, sections were incubated overnight at 4 °C with primary antibodies (Supplementary Data 6) diluted in 0.8% BSA in TBS. After washing, slides were incubated for 1 h at RT with the corresponding secondary antibodies (Supplementary Data 6), also diluted in TBS. A final TBS wash was followed by embedding with Aqua PolyMount (#18606, Polysciences Europe GmbH), and coverslips were applied. Antibody specificity was verified by processing control sections identically, but omitting the primary antibody. All sections from a given subject were mounted on a single slide and stained in the same batch to ensure consistency. Images were acquired using an LSM 510 Meta confocal laser scanning microscope equipped with a Plan-Apochromat 63×/1.4 oil DIC objective (Zeiss GmbH).

### Muscle proteome sample preparation

Approximately 10 µg of each sample was used for shotgun analysis. The samples were carbamidomethylated by incubating with 10 mM of Dithiothreitol (DTT) (#43819, Merck) for 30 min at 37 °C, followed by adding 50 mM Chloroacetamide (CAA) (#A15238, Alfa Aesar) at RT for 30 min in the dark. The samples were then quenched using 50 mM DTT at RT for 20 min. They were then purified by adding a mixture of carboxyl-coated paramagnetic SP3 beads (#45152105050250 and #65152105050250, Cytiva) in 1:1 (vol/vol) ratio. 1 µL of the SP3 beads was added to the sample with HPLC-quality ethanol to a final concentration of 80%. The samples were incubated in ethanol for 20 min and then washed with 90% HPLC-quality acetonitrile and digested with Trypsin (#37286, Serva)/Lys-C at a weight ratio of 1:100 and 1:50 enzyme to protein, respectively, in 50 mM HEPES-NaOH buffer (pH 7.5) supplemented with 2.5 mM CaCl_2_ (#C3306, Sigma). After overnight digestion, the peptides were acidified using formic acid to a pH <3 and purified using C18 stage tips^70^. The stage tips were prepared by packing a 200 µL stage tip with small punched-out discs of C18 material (Empore cation extraction disks #66889-U, Merck). The tips were first washed with 50 µL of 100% HPLC-quality methanol (Lichrosolv #1.06035.2500, Supelco, Merck), followed by 50 µL of 100% HPLC-quality acetonitrile (Lichrosolv #1.00029.2500, Supelco, Merck). Then, the tips were equilibrated with 50 µL of 0.1 % formic acid (Lichropur #5.43804.0100, Merck) in HPLC water. The samples were loaded, washed with 50 µL of 0.1% (vol/vol) formic acid in HPLC water, and eluted with 40 µL of 50% acetonitrile. The stage tipping steps were carried out in a centrifuge at 2000 × *g* for 1 to 2 min. The eluted samples were then dried in a vacuum concentrator and resuspended in 0.1% formic acid in HPLC water.

### Phosphopeptide enrichment

300 µg of the proteome sample was used for phosphopeptide enrichment. The samples were carbamidomethylated and digested using the Trypsin/LysC mixture (Promega) as described above but without calcium chloride in the digestion buffer. The samples were then desalted using HR-X SPE columns (Macherey Nagel) using similar conditions as the stage tipping procedure mentioned above but with 400 µL of solution and centrifugation at 50 × *g* for 1 min at RT. The desalted peptides were dried in a SpeedVac vacuum concentrator and resuspended in 250 µL of binding buffer (80% Acetonitrile, 0.2% Glycolic acid, 5% TFA) for 5 min at 25 °C in a thermoshaker at 250 rpm. To the dissolved peptides, Ti-NTA magnetic beads (#74302, Cube Biotech) resuspended in binding buffer were added at 10× the peptide amount. Phosphopeptides were enriched following the manufacturer's protocol, dried in the SpeedVac and desalted using self-packed SDB-RPS stage tips when necessary. The stage tipping procedure until sample loading was similar to C18; after loading the sample, it was washed with 0.1% FA in HPLC water and 80% ACN acidified with 0.1% FA. The samples were then eluted with 80% ACN with 1% Ammonium hydroxide. The eluted samples were then dried in a vacuum concentrator and resuspended in 0.1% formic acid in HPLC water.

### Functional studies in murine myotubes

C2C12 myoblasts were cultured in high glucose DMEM containing L-glutamine, supplemented with 15% fetal calf serum (FCS), 2 mM non-essential amino acids, 1 mM sodium pyruvate and 100 μ/mL penicillin as well as 100 μ/mL streptomycin (PS) (proliferation medium) at 37 °C and 5% CO_2_ to a confluency of approximately 70%. Differentiation was induced by changing the medium to DMEM supplemented with 2% horse serum, 2 mM non-essential amino acids, 1 mM sodium pyruvate and PS (differentiation medium). At the same day, cells were transfected with control siRNA (Mission siRNA universal negative control, #SIC001, Sigma-Aldrich) or siRNA directed against murine BAG3, HSPB1, PDLIM3, PLIN5 and XIRP1 (Supplementary Table 3) as indicated, using JetPRIME transfection reagent (101000001, Polyplus). On the second day of differentiation, siRNA transfection was repeated. On the fifth day of differentiation, cells were treated with BafA1 (300 nM; sc-201550A, Santa Cruz Biotechnology) for 7 h prior to cell lysis and analysis by SDS-PAGE and immunoblotting. To investigate regulation of the CASA system under acute, damage-inducing mechanical stress, differentiated myotubes were subjected to damage EPS for 7 h as previously described^8^. During EPS treatment, myotubes received 300 nM BafA1 or a similar amount of solvent, followed by cell lysis and analysis by SDS-PAGE and immunoblotting. Quantification of blot lane intensities was performed using ImageJ. Values were normalized for loaded protein amount deduced by Ponceau S staining. Data were analyzed for statistical significance using GraphPad Prism v.10.

### Proximity biotinylation

To generate C2C12 myoblasts that stably express TurboID-BAG3, cDNAs encoding full-length human BAG3 and V5-TurboID-NES were amplified by PCR using the plasmids pCMV-Tag2B-BAG3^11^ and V5-TurboID-NES-pcDNA3 (a kind gift of Christian Behrends), respectively, as templates, followed by subcloning into pLenti-SV40-EGFP. The resulting plasmid pLenti-SV40-TurboID-BAG3 was then used to produce lentiviral particles in HEK293T cells. HEK293T cells were grown in proliferation medium (high glucose DMEM containing L-glutamine supplemented with 10% fetal calf serum (FCS), 2 mM non-essential amino acids, 1 mM sodium pyruvate, 100 μ/ml penicillin and 100 μ/ml streptomycin) at 37 °C and 5% CO_2_ to a confluency of 60% and transfected with JetPRIME transfection reagent (101000001, Polyplus) to introduce a second-generation lentiviral packaging system encompassing the plasmids pLenti-SV40-TurboID-BAG3, pMD2.G and psPAX2. After 48 h incubation, the transfected HEK293T were supplemented with culture media containing 30% FCS to induce production of lentiviral particles. Again 48 h later, lentiviral particles were harvested from the cell culture supernatant by centrifugation at 800 × *g* and filtering through a 0.45 µm mesh. C2C12 myoblasts were grown to a confluency of 40%, inoculated with lentiviral particles for 24 h and stably transduced cells selected with puromycin (1–2 µg/mL). C2C12 myoblasts stably expressing TurboID-BAG3 were differentiated to mytubes for 7 days, 50 µM biotin was added for 1.5 h at 37 °C, followed by harvesting and processing for mass spectrometry.

Samples were dissolved in lysis buffer (2% SDS in 50 mM HEPES, pH 7.5), heated to 95 °C for 5 min and further lysed by 3 cycles of 30 s sonication, 30 s pause in a Bioruptor device (Diagenode). Cell debris was removed by 20 min centrifugation at room temperature and 1 mg of protein was fourfold diluted with 50 mM HEPES buffer. To enrich biotinylated proteins, 75 µL of washed Streptavidin-Sepharose slurry was added and incubated on a tumbling tube rotator overnight at 4 °C. The beads were sequentially washed with 500 µL each of (1) RIPA buffer, (2) RIPA buffer, (3) 1 M KCl, (4) 0.1 M Na2CO3, (5) 2 M Urea in 10 mM Tris-HCl pH8.0, (6) RIPA buffer, (7) RIPA buffer, (8) 0.1 M TEAB pH 8.0, (9) 0.1 M TEAB pH 8.0 and (10) 2 M Urea in 0.1 M TEAB pH 8.0. Next, the beads were incubated with 300 µL of 5 mM DTT in 4 M Urea, 100 mM TEAB pH 8 at 37 °C for 1 h to denature proteins, followed by incubation with 30 mM acrylamide for 40 min at room temperature. 0.65 µg of LysC in 4 M Urea/100 mM TEAB, pH 8 were added, incubated at 37 °C for 6 h before dilution with 100 mM TEAB to a final concentration of 1.6 M urea. Next, 1.65 µg of trypsin was added and incubated at 37 °C overnight; beads were pelleted by centrifugation at 800 × *g* for 1 min and the supernatant was transferred into a fresh tube. The beads were washed with 300 µL of 5% ACN and 0.5% AcOH, and the supernatants were merged, acidified with 1% formic acid and desalted with self-packed C18 stage tips^70^. The desalted peptides were resuspended in 60 µL in 5% ACN and 5% FA buffer, sonicated for 5 min and centrifuged at 20,000 × *g* for 5 min at room temperature.

### In-vitro binding and ubiquitylation studies

For expression and purification of PDLIM3, the cDNA encoding human PDLIM3 was PCR amplified using pCMV6-PDLIM3-Myc-FLAG (#RC212972, Origene) as template, and subcloned into plasmid pMAL-c2 (New England Biolabs). The resultant construct was used for expression of MBP fusion proteins in *E. coli* TG1 cells, followed by purification on an amylose resin according to the manufacturer’s protocol (New England Biolabs). BAG3 encoding cDNA, subcloned into pET-M11 (Novagene), was used for expression of HIS-fusion proteins in *E. coli* BL21(DE3) and purified on Ni-NTA-agarose as described by the manufacturer (Qiagen). Prior to final elution, isolated complexes were incubated with 2 mM ATP, 1 mM MgCl_2_, 20 mM MOPS, pH 7.2, 100 mM KCl to dissociate bacterial HSP70. Interaction studies were performed on Ni-NTA-agarose in 20 mM MOPS, pH 7.2, 100 mM KCl, 40 mM imidazol (binding buffer) containing 1 µM BAG3. A preparation of MBP-PDLIM3 that also contained equimolar amounts of MBP due to proteolysis during expression and purification was used at 1.5 µM concentration (MBP-PDLIM3/MBP). Following incubation for 1 h on ice, samples were centrifuged through Mobicol columns (#M1003, MoBiTec) to collect the Ni-NTA-Agarose. Beads were washed 3-times with binding buffer followed by elution with buffer containing 200 mM imidazol. Samples were analyzed by SDS-PAGE and immunoblotting.

CASA-mediated ubiquitylation was in-vitro reconstituted with purified components. A cytoskeleton fraction was derived from C2C12 myotubes, differentiated for 5 days. Cells were collected from one 10 cm culture dish and lysed in RIPA buffer without SDS (25 mM Tris-HCl, pH 8.0, 150 mM NaCl, 0.5% sodium deoxycholate, 1% Nonidet P-40, 10% glycerol). A post-nuclear supernatant (PNS) was obtained by centrifugation at 3000 × *g* for 10 min at 4 °C, followed by cytoskeleton isolation through centrifugation of the PNS at 16,000 × *g* for 10 min at 4 °C. The resultant pellet was resuspended in incubation buffer (20 mM MOPS, pH 7.2, 100 mM KCl, 10 mM ATP, 10 mM MgCl_2_, 10 mM DTT, 0.02 % PMSF). A cytoskeleton fraction derived from 50 µg PNS was added per ubiquitylation reaction, containing 1.8 µM BAG3, 1.8 µM HSC70, 1.8 µM STUB1, 1.8 µM UBCH5B, and 0.6 µM HSP40. When indicated, reactions also contained 0,5 µg/µl ubiquitin and 0,07 µg/µl E1. MBP-PDLIM3 and MBP were added to a final concentration of 1.8 µM as indicated. Samples were incubated for 3 h at RT, followed by SDS-PAGE analysis and immunoblotting.

### Mass spectrometry data acquisition

Purified peptides of human samples were analyzed using an Ultimate 3000 RSLCnano coupled to a Bruker Impact II QTOF mass spectrometer. About 1 µg of the peptide was first loaded to a 2 cm µPAC C18 trap column (Thermo) using a loading buffer of 2% acetonitrile and 0.05% Trifluoro acetic acid at a 12 µL/min flow rate. They were then separated using a 50 cm µPAC C18 microarray pillar column (Thermo) with a gradient of 2 to 30% buffer B (100% Acetonitrile with 0.1% FA) and buffer A (HPLC water, 0.1% FA) over 90 min. The flow rate was set to 600 nL/min, and the column was connected to the mass spectrometer via a Bruker captive spray source with a nano booster. The peptides were measured in DIA mode using Compass HyStar v5.1 (Bruker) with OTOF control v 5.2 (Bruker) and the Chromeleon Plugin v 7.21 (Thermo). The quadrupole was set to filter precursor ions of a set mass window of 25 *m*/*z* with an overlap of 0.5 *m*/*z* on either side of the window. Each cycle consisted of one MS1 acquisition followed by MS2 of 32 windows of 25 *m*/*z*. The MS acquisition rate was set to 4 Hz, leading to a cycle time of 8.25 s. The phosphopeptides were analyzed in DDA mode with the same LC settings. The DDA-MS settings are as follows: the acquisition mass range was set to 200–1750 *m*/*z* at 5 Hz. The top 14 ions were selected for MS2, and the MS2 rate was set to a dynamic mode of 5 to 20 Hz with a target TIC of 25,000 counts, leading to a total cycle time of 0.9 to 3 s. The collision energy was set to 23 to 48 eV depending on the precursor *m*/*z* with an RF value of 1500 Vpp. The collision energy and the transfer time were set to step between 100 and 120% of the set value and 110–155.6 µs for 50% of the time, respectively. The preferred charge range was set to 2–6, and active exclusion was set to exclude precursor ions after 1 spectrum for 0.4 min or until the intensity of the ion was 3× the previous intensity. BAG3-proximity biotinylation samples were analyzed with an Ultimate 3000 nano-UHPLC equipped with in-house-produced columns (45 cm fused silica capillaries with 360 μm outer diameter/100 μm inner diameter (fabricated with a P-2000 laser puller, Sutter Instruments) packed with 3 μm ReprosilPur AQ C 18 particles (Dr. Maisch) coupled to an Orbitrap Fusion Lumos mass spectrometer (Thermo). 3 µl of each sample was loaded onto the analytical column at a flow rate of 850 nL/min in 100% solvent A (0.1% FA), followed by separation with a 120 min linear gradient from 5 to 35% solvent B (95% ACN, 0.1% FA) at a flow rate of 300 nL/min. Data was acquired in DIA mode with MS1 scans from *m*/*z* 350−1200 followed by 36 consecutive HCD MS2 scans at 30% collision energy with an isolation window of *m*/*z* 24.1 (overlap of *m*/*z* 0.5). Both MS1 and MS2 scans were acquired in the Orbitrap analyzer with a resolution of 120,000 and 30,000, respectively. For MS1/MS2 scans, the AGC and maximum injection time were set to 5 × 10^5^/1 × 10^6^ and 20 ms/60 ms, respectively, resulting in a total cycle time of 3.44 s.

### Proteome data analysis

The measurement files were analyzed using DIA-NN software^71^ version 1.8. The Bruker raw files (.d/.baf) were first converted to mzML generic format using MSConvertGUI (ProteoWizard^72,73^). For MSConvert, the settings were as follows: Output format – mzML, Binary encoding precision was set to 32-bit, TPP compatibility, and Zlib compression were unchecked. The peak picking filter was assigned to the default vendor msLevel and was set as the top filter. Human skeletal muscle-specific proteins were selected from the Human Protein Atlas, and FASTA sequences for these proteins were downloaded from UniProt using the retrieve ID function (May 2022, 12,959 entries). The converted files and the FASTA file were loaded to the DIA-NN software, and the following settings were applied: FASTA digest for library-free search and Deep learning-based prediction was switched on. Protease was set to Trypsin/P with one missed cleavage and one variable modification (oxidized methionine). N-terminal M excision and cysteine carbamidomethylation were selected as fixed modifications. The peptide length was set to 7–35, and the charge was set to 1–4. The precursor and the fragment *m*/*z* range was set to 200–1800. MS1 and MS2 mass accuracy were set to 25 PPM and the scan window to 2. Each fraction (pellet and supernatant) was analyzed separately, with an independent search of all combined files conducted to assess efficacy of fractionation. MBR was switched on, and quantification was based on genes. The neural network classifier was set in double-pass mode, and the library generation mode was set to smart profiling. DIA-NN was set to create a quantities matrix containing quantifications for the proteins identified in each replicate, with one column for each replicate. After DIA-NN analysis, the datasets were filtered to include only proteins with 70% valid values in at least one group. LIMMA-moderated *t*-test with a BH FDR correction was used to determine significance of changes in protein abundance between conditions^74^.

### Phosphoproteome data analysis

The DDA measurements from the phosphopeptide-enriched samples were first converted to mzM using MsConvert. The settings were as follows: Output format was set to mzML, write index, binary encoding precision was set to 64 bits, and TPP compatibility and Zlib compression were checked. The peak-picking filter was set to vendor MS level. The converted data were queried using Fragpipe V 18.0 containing Msfragger V 3.5^75^, Philosopher V 4.4.0^76^, Ionquant V 1.8.0^77^, and Percolator V 3.05.0^78^. LFQ-Phospho workflow was loaded, and the skeletal muscle-specific FASTA database and reverse sequences were added. The resulting STY.tsv file was further processed using the Perseus software package^79^ v1.6.15.0. The matrix was log2-transformed, followed by grouping according to time points and filtering for 70% valid values in at least one group. Missing values were replaced from the lower end of the normal distribution with a width of 0.3 and a downshift of 1.3. The time points were compared using an ANOVA-based multiple-sample test. Phosphosites with a significance *p*-value < 0.05 were filtered, and significant pairs were determined using a post-hoc test with a significance *p*-value < 0.05. The raincloud was made using the Raincloud-shiny app from Allen et al.^80^. The comparisons in the boxplot are based on the *p*-value from the paired *t*-test provided in the Shiny app.

### Proximity biotinylation data analysis

Thermo.raw files were processed using Spectronaut software from Biognosys (v19.5.24116.62635) in the directDIA mode. In particular, 3 to 6 fragment ions for each peptide with the Pulsar search engine incorporated in Spectronaut and UniProt Mus musculus (released 07-01-2022 with 17,090 entries). For database searching, the following settings were applied: trypsin/P (with 2 allowed missed cleavages); fixed modification: propionamide at cysteine; variable modifications: acetylation at protein N-termini and oxidation of methionine. Next, the false discovery rate (FDR) for peptide and protein identification was set to 1%. For peptide/protein quantification, a *q*-value cutoff of 0.01 and 0.05, respectively. No normalization strategies were applied. The dataset was then constricted to proteins reliably quantified in all three BAG3-turboID biotin samples with a raw intensity >100. For control samples, we then applied a two-step and category-based imputation approach. First, proteins quantified in no or only one of the control samples were imputed from a minimum-probability Gaussian distribution (center defined by the minimal quantile of 0.05 of the intensity distribution, dispersion set from the median protein-wise SD scaled by sigma =1). Then, the missing values for proteins quantified in two control replicates were imputed using the impSeqRob algorithm from the rrcovNA R package^81,82^. A 50% change in protein abundance in the biotin compared to control samples supported by a limma-moderated *p*-value < 0.05 was considered significant.

### Data visualization

The boxplots and raincloud plots with proteomics data were made using the Raincloud-shiny app^80^ as well as the R-program in R-Studio. We used R version 4.4.2^83^ and R Studio version 2023.6.1.524^84^ and the following R packages: Cairo v. 1.6.2^85^, cowplot v. 1.1.3^86^, EnhancedVolcano v. 1.22.0^87^, ggplotify v. 0.1.2^38^, ggpubr v. 0.6.0^88^, gridExtra v. 2.3^89^, limma v. 3.60.6^74^, patchwork v. 1.3.0^90^, png v. 0.1.8^91^, RColorBrewer v. 1.1.3^92^, readxl v. 1.4.5^93^, svglite v. 2.1.3^94^, tidyverse v. 2.0.0^95^, writexl v. 1.5.2^96^. Protein illustrations with their domains and phosphosites were made using IBS Illustrator version 2^97^. Some of the figure panels (Figs. 1a, g and S1–S3) were arranged and icons were created using BioRender.com. Proximity biotinylation data were visualized with Instant Clue (v0.12.2)^98^., ggpubr v. 0.6.0^88^, gridExtra v. 2.3^89^, limma v. 3.60.6^74^, patchwork v. 1.3.0^90^, png v. 0.1.8^91^, RColorBrewer v. 1.1.3^92^, readxl v. 1.4.5^93^, svglite v. 2.1.3^94^, tidyverse v. 2.0.0^95^, writexl v. 1.5.2^96^. Protein illustrations with their domains and phosphosites were made using IBS Illustrator version 2^97^. Some of the figure panels (Figs. 1a, g and S1–S3) were arranged and icons were created using BioRender.com. Proximity biotinylation data were visualized with Instant Clue (v0.12.2)^98^. Venn Diagrams were generated in BioVenn^99^.

### Reporting summary

Further information on research design is available in the Nature Portfolio Reporting Summary linked to this article.

## Supplementary information

Supplementary Information File

Descriptions of Additional Supplementary Files

Supplementary Data 1

Supplementary Data 2

Supplementary Data 3

Supplementary Data 4

Supplementary Data 5

Supplementary Data 6

Reporting Summary

Transparent Peer Review file

## Source data

Source Data

## Data Availability

The mass spectrometry proteomics data have been deposited to the ProteomeXchange Consortium^100^ via the PRIDE partner repository^101^ with the dataset identifiers PXD044976 for the human fractionated muscle proteome and phosphoproteome and PXD074000 for the BAG3 proximity biotinylation data. All other data generated in this study are provided in the Source Data file. Source data are provided with the paper. Source data are provided with this paper.

## Code availability

The R-script for two-step imputation used to analyse the proximity biotinylation data is provided at https://github.com/MiguelCos/BAG3_turboID_proteomics-Differential-abundance-analysis.
