# Supplementary material for: Fractionated proteomics identifies a protein network mitigating resistance exercise-induced damage in human skeletal muscle
Source: Nat Commun. 2026 Jul 28;17:7110. doi: 10.1038/s41467-026-75501-y (PMC13415819; doi:10.1038/s41467-026-75501-y)
Supplement: Supplementary file 1 — Supplementary Information File [file 41467_2026_75501_MOESM1_ESM.pdf]

Supplementary Information

Fractionated proteomics identifies a protein network mitigating resistance exercise-induced damage in human skeletal muscle

Maithreyan Kuppusamy[1,2,#], Daniel Jacko[3,4,#], Yamini Gupta[5,#], Sandro Sieger[5], Kirill Schaaf[3,4], Martin Matijass[1,3,6], Käthe Bersiner[7], Jonas Zacher[8], Sara Bonini[2], Miguel Cosenza-Contreras[9], Peter F.M. van der Ven[5], Wilhelm Bloch[3], Dieter O. Fürst[5], Dominic Winter[2], Jörg Höhfeld[5,$,*], Pitter F. Huesgen[1,9,10,$,*], Sebastian Gehlert[3,7,$,*]

[1] Central Institute for Engineering, Electronics and Analytics, ZEA-3, Forschungszentrum Jülich, Germany

[2] Research Centre One Health Ruhr, University Alliance Ruhr & University Hospital Essen Medical Faculty, University Duisburg-Essen, Essen, Germany

[3] Department of Molecular and Cellular Sports Medicine, Institute of Cardiovascular Research and Sports Medicine, German Sport University Cologne, Cologne, Germany

[4] Olympic Base Centre, North Rhine-Westphalia/Rhineland, Cologne, Germany

[5] Institute for Cell Biology, Rheinische Friedrich-Wilhelms University Bonn, Bonn, Germany,

[6] Current address: Institute of Biochemistry, Department for Chemistry, University of Cologne, Cologne, Germany

[7] Department for the Biosciences of Sports, Institute of Sports Science, University of Hildesheim, Hildesheim, Germany

[8] Department of Preventative and Rehabilitative Sports and Performance Medicine, Institute of Cardiology and Sports Medicine, German Sports University Cologne, Cologne, Germany

[9] Faculty of Biology, University of Freiburg, Freiburg, Germany

[10] CIBSS- Centre for Integrative Biological Signaling Studies, University of Freiburg, Freiburg, Germany

[#] These authors contributed equally: M.K., D.J., Y.G.

[$] These authors jointly supervised this work: J.H., P.F.H., S.G.

* Correspondence: Cell biology: J. Höhfeld, hoehfeld@uni-bonn.de;

Proteomics: P. F. Huesgen, pitter.huesgen@biologie.uni-freiburg.de;

Cohort and physiology: S. Gehlert, gehlert@uni-hildesheim.de

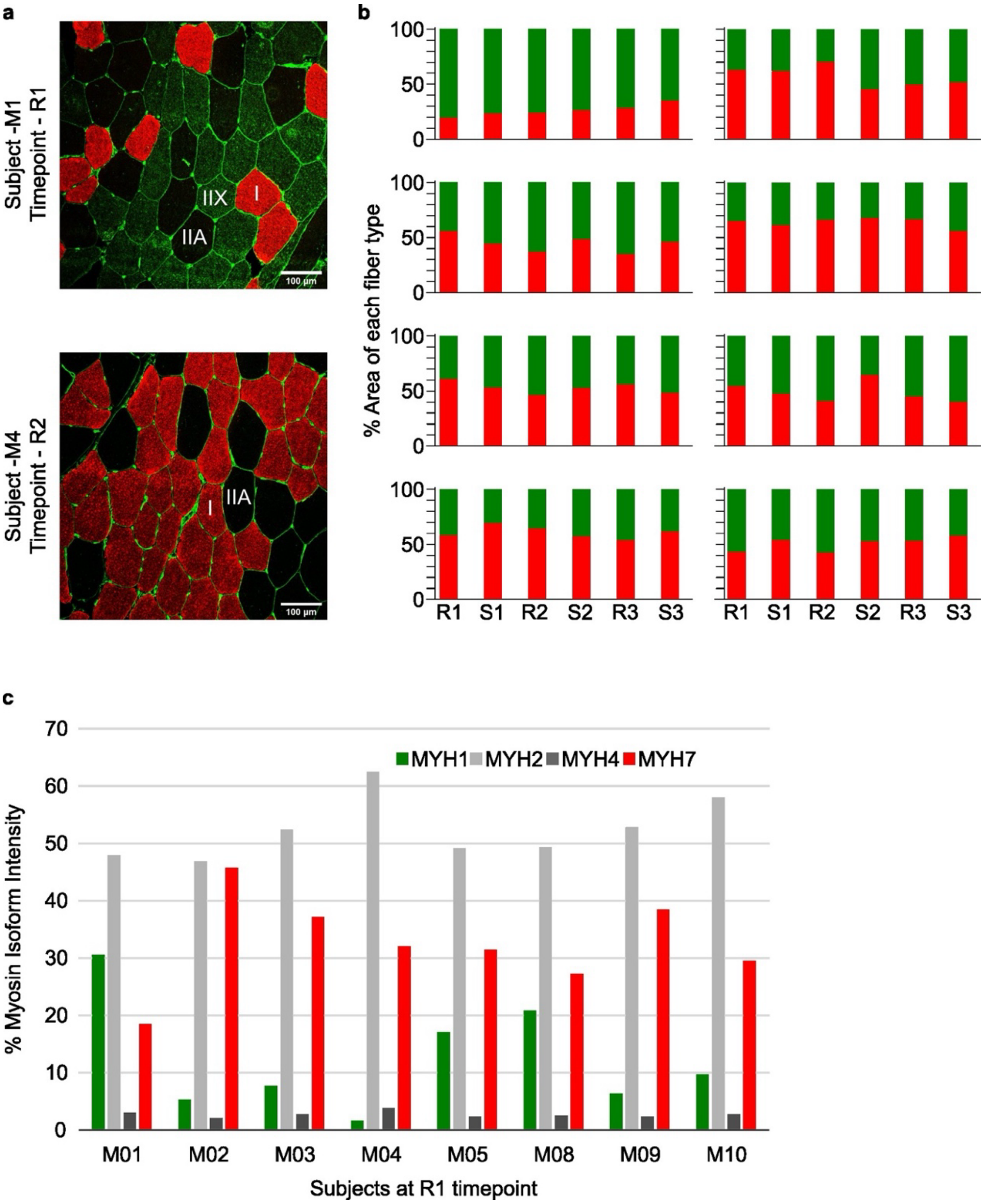

Supplementary Figure 1. Assessment of fibre type composition. **a** Immunostaining for myosin heavy chain isoforms. MyHC-I (MYH7), representing Type 1 fibers, is stained red; MyHC-II-X (MYH1), representing Type 2B fibers, is stained green; and MyHC-II-A (MYH2), representing Type 2A fibers, is unstained. **b** Proportion covered by each fiber type in the immunostainings. The red bars represent type 1 fibers, and the green bars represent type 2 A and B combined. Analysed section area is normalized to 100%. **c** Mass spectrometry-based quantification of myosin heavy chain

isoforms at timepoint R1. MaxQuant LFQ intensities are summed and normalized to 100 percent.

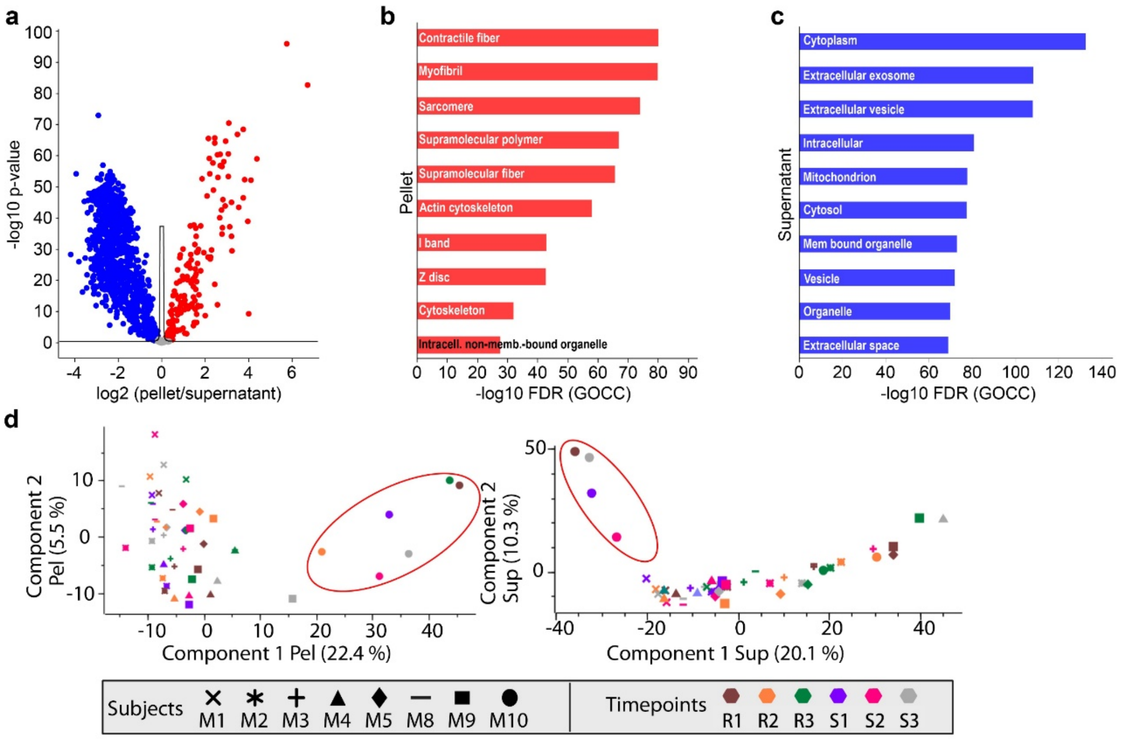

Supplementary Figure 2. Comparison of the proteomes in Triton X-100-fractionated muscle biopsies. **a** Volcano plot comparing protein abundance in the pellet and supernatant fractions. Red and blue dots indicate proteins that are significantly more abundant (FDR-controlled t-test <0.05, S0 = 0.1) in the pellet and supernatant fractions, respectively. Gene ontology terms (cellular component) enriched among proteins with significantly higher abundance in **b** the supernatant fraction (blue) and **c** the pellet fraction (red). The top 10 GO-CC terms are shown. **d** Principal Component Analysis of proteins quantified in the pellet (left panel) and the supernatant (right panel) fractions of all eight subjects and six timepoints. M10 is the only female participant.

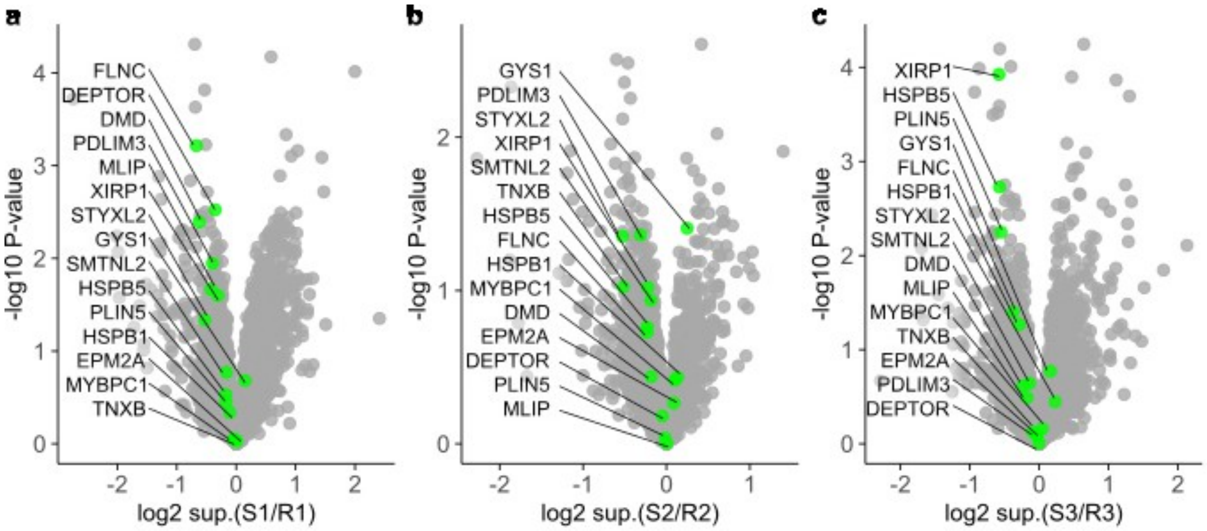

Supplementary Figure 3. Volcano plots comparing protein abundance in supernatant fractions after SMO (S) and rest (R) in **a** the untrained state (S1/R1), **b** the adapted state (S2/R2), and **c** the deadapted state (S3/R3). Y-axis depicts -log10 (LIMMA-moderated T-test p-value). Note that none of the observed changes in the supernatant are significant after adjusting for multiple hypothesis testing. Proteins with significant changes in abundance in the pellet fraction are highlighted in green.

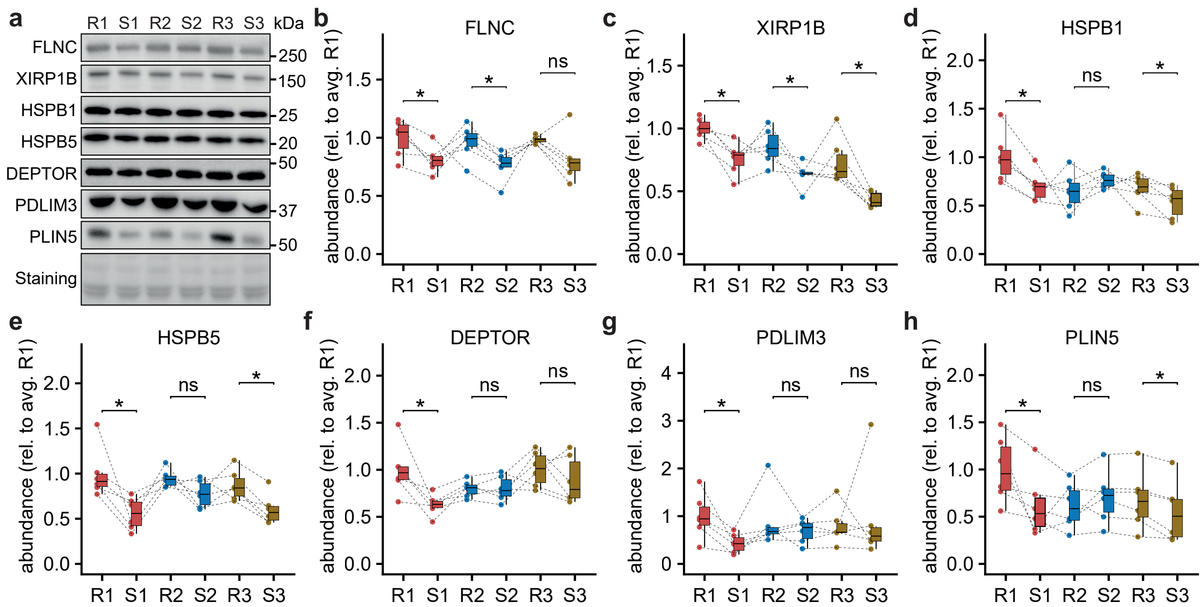

Supplementary Figure 4. Immunoblot analysis of protein abundance in the supernatant fractions. **a** Exemplary immunoblots of FLNC, XIRP1B, HSPB1, HSPB5, DEPTOR, PDLIM3 and PLIN5. Quantitative analysis of immunoblots with antibodies detecting **b** FLNC, **c** XIRP1B (XIRP1A was not detected at this exposition time), **d** HSPB1, **e** HSPB5, **f** DEPTOR, **g** PDLIM3 and **h** PLIN5 immunoblots in the supernatant fraction in the stratified cohort (n=6). * Wilcoxon matched-pairs signed rank test p-value ≤ 0.05. **b-h** Box plots illustrate the distribution of data, with the line within each box representing the median. Box plot boundaries define the interquartile range (IQR), encompassing the 25th and 75th percentiles. Whiskers extend to the minimum and maximum data points within 1.5 times the IQR.

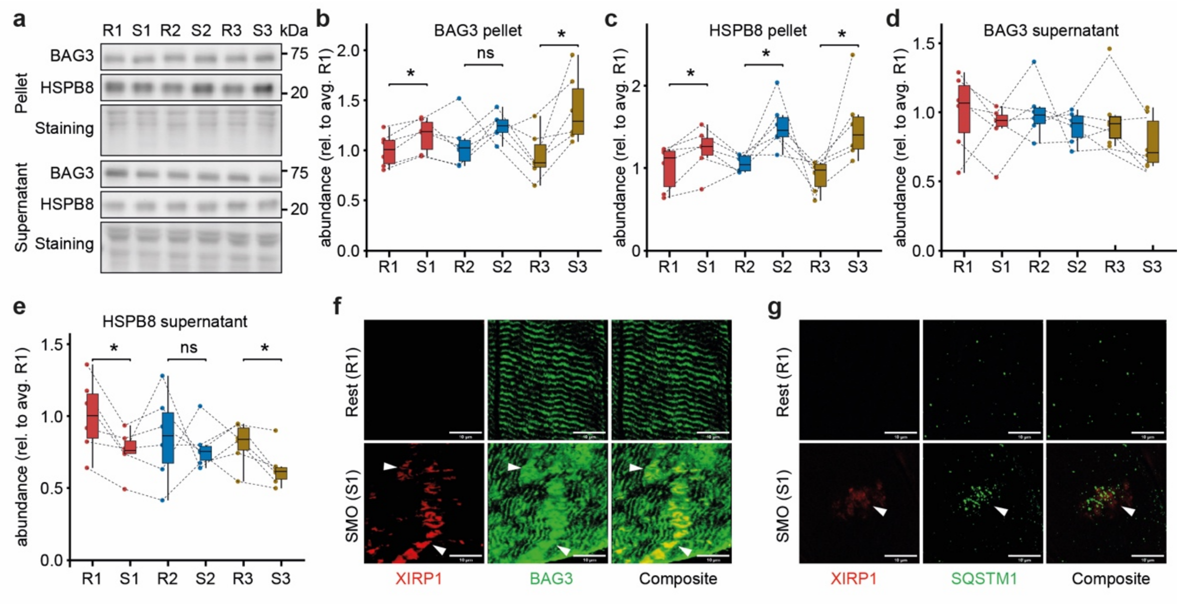

Supplementary Figure 5. Immunoblot and immunohistochemical analysis of CASA complex components. **a** Exemplary immunoblots of BAG3, HSPB8 in the pellet and supernatant fractions with stained membranes shown as loading controls. Quantification of **b** BAG3 and **c** HSPB8 in the supernatant fraction, **d** BAG3 and **e** HSPB8 in the pellet fraction. n = 6 (stratified cohort), normalized to abundance at R1. Box plots illustrate the distribution of data, with the line within each box representing the median. Box plot boundaries define the interquartile range (IQR), encompassing the 25th and 75th percentiles. Whiskers extend to the minimum and maximum data points within 1.5 times the IQR.* Wilcoxon matched-pairs signed rank test p-value ≤ 0.05. Co-immunostainings for **f** XIRP1 and BAG3 and **g** XIRP1 and SQSTM1 before (R1) and after SMO (S1) in untrained muscle. The scale bars indicate 10 µm.

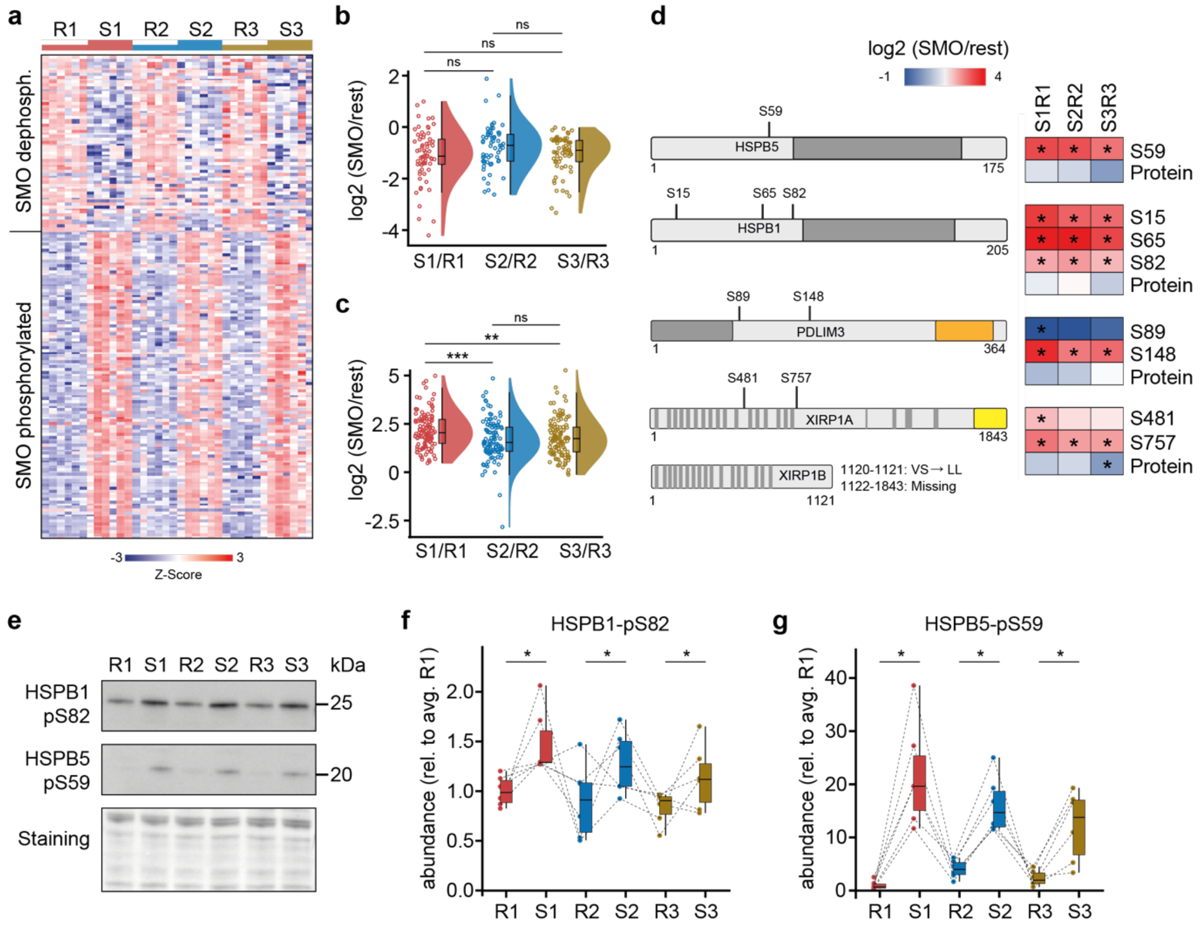

Supplementary Figure 6. Analysis of SMO-regulated changes in the phosphoproteome in the supernatant. **a** Hierarchical clustering of Z-score normalized intensities of phosphosites significantly changing in the supernatant (ANOVA test p-value < 0.05, followed by Tukey´s post-hoc test with FDR < 0.05). Raincloud plots show the distribution of regulated phosphosites that are significantly **b** dephosphorylated and **c** phosphorylated in response to SMO in untrained (S1/R1), adapted (S2/R2), and deadapted (S3/R3) conditions. **d** Regulated phosphosites in selected soluble proteins that showed increased association with the cytoskeleton after SMO. Change in abundance is indicated by color scale, * marks time points with significant changes in phosphorylation or protein abundance (ANOVA test p-value < 0.05, followed by Tukey´s post-hoc test with FDR < 0.05. Yellow highlights the FLNC-binding domain of XIRP1, orange the LIM domain of PDLIM3. Dark grey boxes indicate the a-crystallin domain in HSPB1 and 5, the PDZ domain in PDLIM3, and grey lines indicate the XIN repeats in XIRP1. Created in BioRender. Kuppusamy, M. (2026) https://BioRender.com/ttusjik **e** Exemplary immunoblots for HSPB5 phosphorylation at Ser59 (HSPB5-pS59) and HSPB1 phosphorylation at Ser82 (HSPB1-pS82) in the

supernatant fraction. Quantification of **f** HSPB1-pS82 and **g** HSPB5-pS59 immunoblots with the supernatant fraction in the stratified cohort (n=6). Box plots illustrate the distribution of data, with the line within each box representing the median. Box plot boundaries define the interquartile range (IQR), encompassing the 25th and 75th percentiles. Whiskers extend to the minimum and maximum data points within 1.5 times the IQR. *Wilcoxon matched-pairs signed rank test p-value ≤ 0.05.

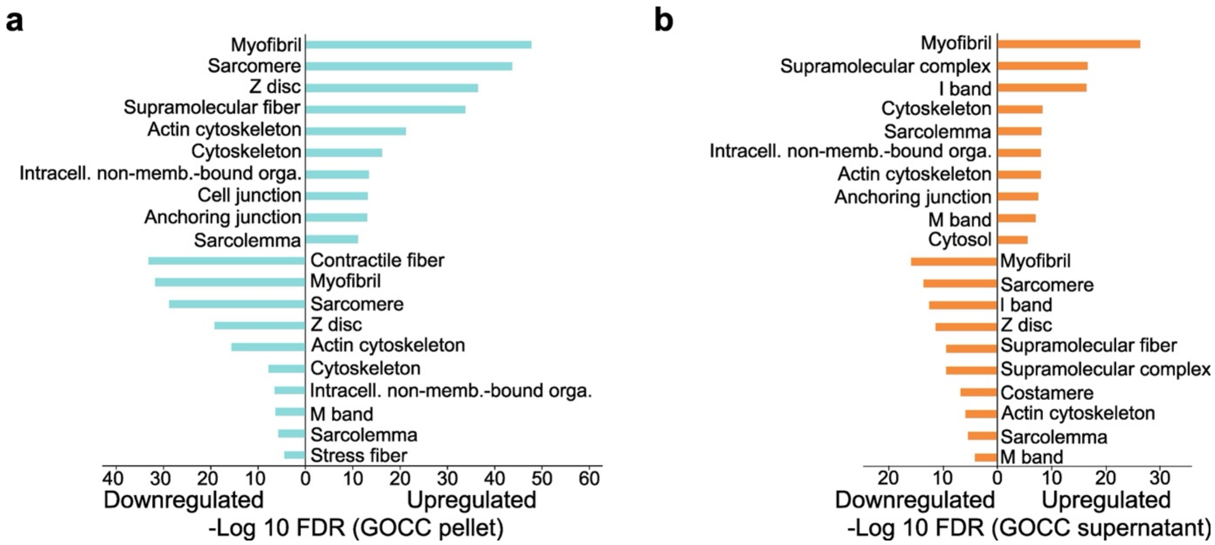

Supplementary Figure 7. Gene ontology cellular component (GO-CC) terms associated with proteins with SMO-regulated phosphosites in **a** the pellet and **b** the supernatant fractions.

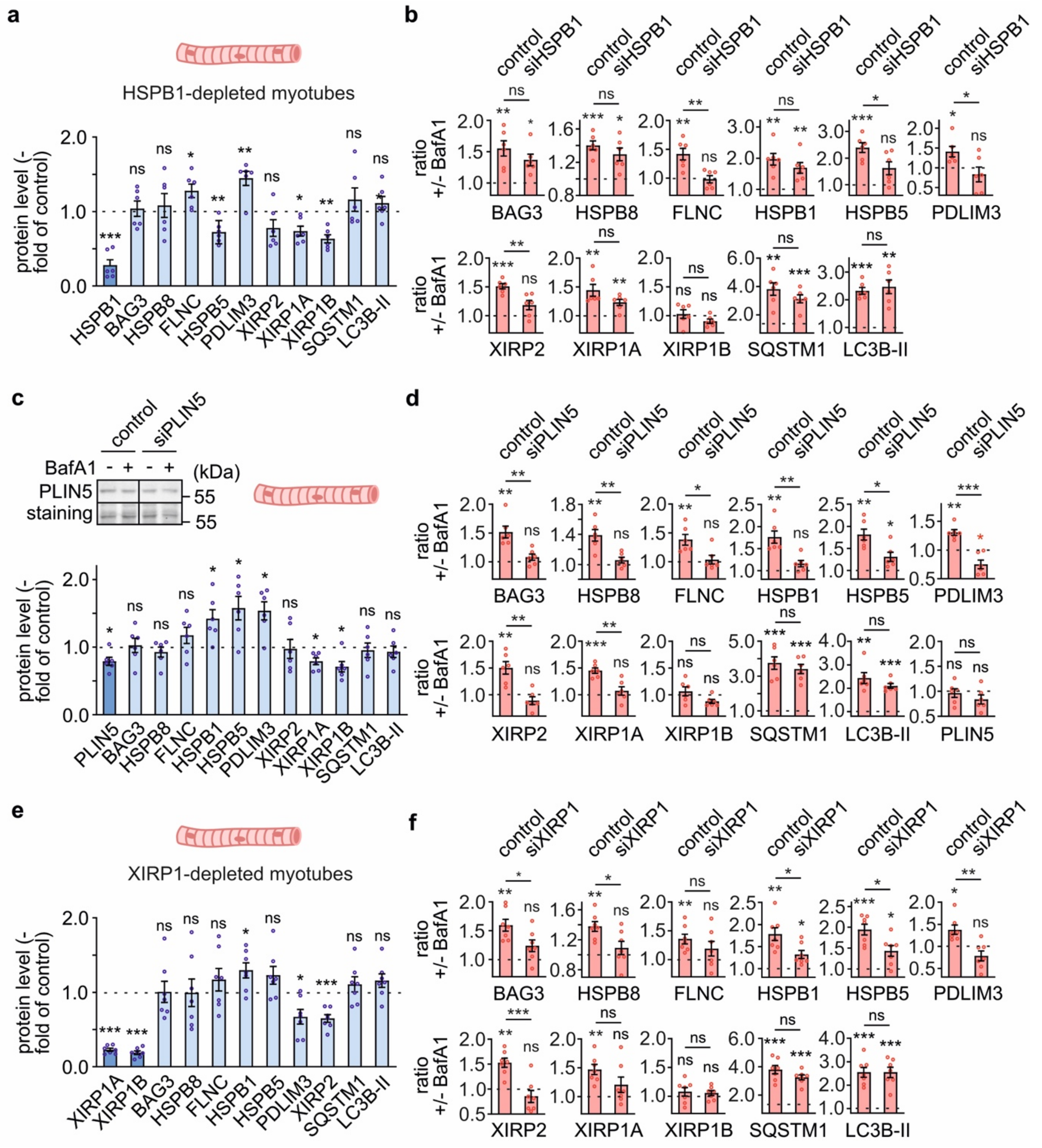

Supplementary Figure 8. CASA activity in C2C12 myotubes depleted for RE-responsive proteins. Quantification of **a, c, e** protein abundance and **b, d, f** protein turnover of the CASA complex components BAG3 and HSPB8, the CASA substrate FLNC, the RE-responsive proteins HSPB1, HSBP5, PDLIM3, PLIN5, XIRP1A, XIRP1B and XIRP2, the autophagy receptor SQSTM1 and LC3B-I and LC3B-II as autophagy markers in **a, b** HSPB1-depleted, **c, d** PLIN5-depleted and **e, f** XIRP1-depleted myotubes based on immunoblots shown in Fig. 4b. Depletion of PLIN5 was verified in **c** by immunoblotting of cell lysates derived from myotubes transfected with

control siRNA (control) or PLIN5 targeting siRNA (siPLIN5). A Ponceau S-stained gel band is shown as a loading control (staining). When indicated, myotubes were treated with BafA1 prior to cell lysis for 7 h. Protein level in control cells was set to 1 and is represented as a dashed line in **a, c, e**. In **b, d, f** the dashed line represents a value of 1 indicating no change in protein abundance between BafA1-treated and untreated cells. Data are shown as mean values +/- SEM, n = 6 in **a, b, c, d** and n = 7 in **e, f**. Statistical analysis was carried out using two-tailed unpaired t-test with Welch's correction: *p < 0.05, **p < 0.01, ***p < 0.001, ns – non-significant. Asterisks above column bars indicate significance compared to control. Asterisks above column-connecting lines indicate significance between the compared samples.

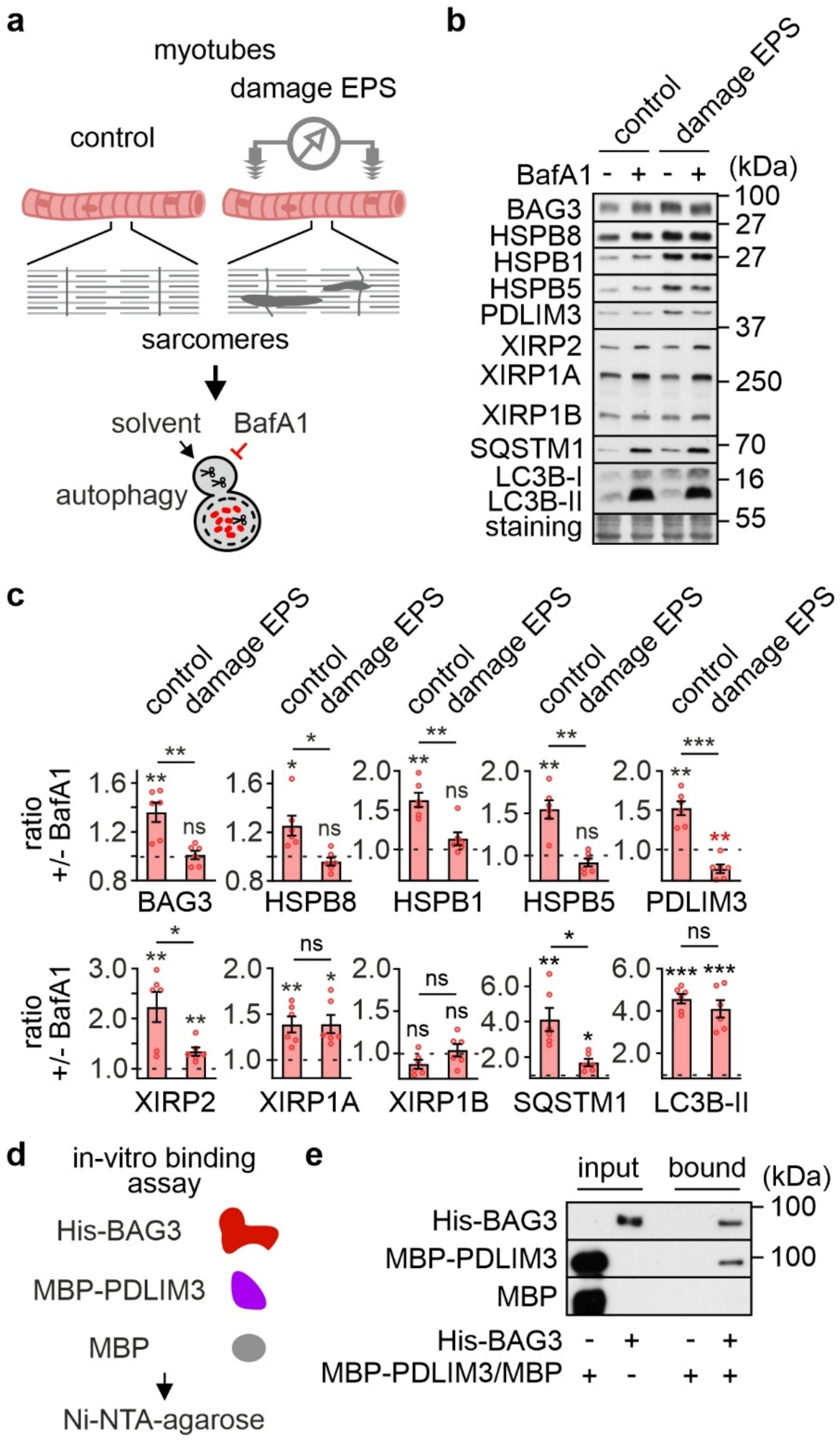

Supplementary Figure 9. CASA activity in C2C12 myotubes subjected to damage-inducing electrical pulse stimulation (damage EPS) and interaction studies with purified BAG3 and PDLIM3. **a** C2C12 myotubes were subjected to damage EPS for 7 hours. At the same time cells were treated with 300 nM BafA1 or solvent as indicated. **b** Cell lysates were subjected to SDS-PAGE analysis and immunoblotting using

specific antibodies against the indicated proteins. Ponceau S membrane staining is shown as a loading control (staining). **c** Autophagic protein turnover was quantified by determining the protein abundance with and without BafA1 treatment as obtained under **b**. The dashed line represents a value of 1, indicating no change in protein abundance between BafA1-treated and untreated cells. Data are shown as mean values +/- SEM, n = 6. Statistical analysis was carried out using two-tailed unpaired t-test with Welch's correction: *p ≤ 0.05, **p ≤ 0.01, ***p ≤ 0.001, ns – non-significant. Asterisks above column bars indicate significance compared to control. Asterisks above column-connecting lines indicate significance between the compared samples. Red asterisks indicate a significant reduction of PDLIM3 in damage EPS-treated myotubes upon BafA1 treatment, pointing to a non-autophagic and BafA1-induced degradation pathway for PDLIM3 under acute, lesion-inducing mechanical stress. **d** Schematic representation of the in-vitro binding experiment, monitoring the retention of MBP-PDLIM3 and MBP on NiNTA-agarose in the presence or absence of Histidin-tagged BAG3 (His-BAG3). **e** Following affinity chromatography, the indicated proteins were detected in the input sample (input) and the eluate from the NiNTA-agarose (bound). Input represents 5% of the bound fraction.

Supplementary Table 1. Cohort statistics. * indicates subjects subsequently excluded from analysis of proteome data. BMI: Body Mass Index. SD: Standard Deviation.

Subject	Sex	Age	Height (cm)	Weight (kg)	BMI
1*	M	30	188	82	23,2
2	M	25	186	82	23,7
3	M	20	183	75,5	22,5
4	M	21	183	70	20,9
5	M	29	187	90	25,7
6	M	23	179	89	27,8
7	M	21	182	82	24,8
8*	F	24	153	53	22,6
**Mean** (all)	7 M/ 1F	24,1	180,1	77,9	23,9
**SD** (all)		3,7	11,3	12,0	2,1
**Mean** (stratified)	6 M	23,2	183,3	81,4	24,2
**SD** (stratified)		3,4	2,9	7,7	2,4

Supplementary Table 2. Adaptive and overload resistance exercise regimes.

Exercise	Adaptive Training		Standardized Mechanical Overload (SMO)			
	Reps	Sets	Reps		Sets	
	4	1	4		2	
Leg Extension	8	1	8	(+4/4*)	2	(+1*)
	16	1	16		2	
Leg Press	4	1	4		2	
	8	1	8	(+4/4*)	2	(+1*)
Drop Jumps	10	1	10		2	
Downstairs Walks	182 stairs	1	182 stairs		2	

* Concentric phase of the third set was performed with both legs, excentric phase alternately with one leg

Supplementary Table 3. siRNA used in this study.

siRNA	Nucleotide sequence
siBAG3	CAGGUCAAGUACAAGUCUA[dT][dT]
	CGAAGAGUAUUGACCAAA[dT][dT]
siHSPB1	CGAAGAAAGGCAGGACGAA[dT][dT]
siPDLIM3	CACUCUAAAGCCGAGGCUU[dT][dT]
siPLIN5	CACCCAUCCUGGUGGAACA[dT][dT]
	CCACCUUUGCUGAUGCACA[dT][dT]
siXIRP1	GGAGAAAGCCUGUAAGGUA[dT][dT]
