# Supplementary material for: Fractionated proteomics identifies a protein network mitigating resistance exercise-induced damage in human skeletal muscle
Source: Nat Commun. 2026 Jul 28;17:7110. doi: 10.1038/s41467-026-75501-y (PMC13415819; doi:10.1038/s41467-026-75501-y)
Supplement: Supplementary file 2 — Descriptions of Additional Supplementary Files [file 41467_2026_75501_MOESM2_ESM.pdf]

Description of Additional Supplementary Files

Supplementary Data 1: Table listing proteins identified in the pellet fraction.

Supplementary Data 2: Table listing proteins identified in the supernatant fraction.

Supplementary Data 3: Table listing phosphopeptides identified in the pellet fraction.

Supplementary Data 4: Table listing phosphopeptides identified in the supernatant fraction.

Supplementary Data 5: Table listing proteins identified in the BAG3 proximity biotinylation experiment.

Supplementary Data 6: Antisera and antibodies used in this study.
