# Supplementary material for: Fractionated proteomics identifies a protein network mitigating resistance exercise-induced damage in human skeletal muscle
Source: Nat Commun. 2026 Jul 28;17:7110. doi: 10.1038/s41467-026-75501-y (PMC13415819; doi:10.1038/s41467-026-75501-y)
Supplement: Supplementary file 9 — Reporting Summary [file 41467_2026_75501_MOESM9_ESM.pdf]

nature portfolio

Corresponding author(s): Sebastian Gehlert

Last updated by author(s): May 31, 2026

Reporting Summary

Nature Portfolio wishes to improve the reproducibility of the work that we publish. This form provides structure for consistency and transparency in reporting. For further information on Nature Portfolio policies, see our Editorial Policies and the Editorial Policy Checklist.

Statistics

For all statistical analyses, confirm that the following items are present in the figure legend, table legend, main text, or Methods section.

n/a	Confirmed	
☐	☒	The exact sample size (*n*) for each experimental group/condition, given as a discrete number and unit of measurement
☐	☒	A statement on whether measurements were taken from distinct samples or whether the same sample was measured repeatedly
☐	☒	The statistical test(s) used AND whether they are one- or two-sided<br>*Only common tests should be described solely by name; describe more complex techniques in the Methods section.*
☐	☒	A description of all covariates tested
☐	☒	A description of any assumptions or corrections, such as tests of normality and adjustment for multiple comparisons
☐	☒	A full description of the statistical parameters including central tendency (e.g. means) or other basic estimates (e.g. regression coefficient) AND variation (e.g. standard deviation) or associated estimates of uncertainty (e.g. confidence intervals)
☐	☒	For null hypothesis testing, the test statistic (e.g. *F*, *t*, *r*) with confidence intervals, effect sizes, degrees of freedom and *P* value noted<br>*Give P values as exact values whenever suitable.*
☒	☐	For Bayesian analysis, information on the choice of priors and Markov chain Monte Carlo settings
☐	☒	For hierarchical and complex designs, identification of the appropriate level for tests and full reporting of outcomes
☒	☐	Estimates of effect sizes (e.g. Cohen's *d*, Pearson's *r*), indicating how they were calculated

Our web collection on statistics for biologists contains articles on many of the points above.

Software and code

Policy information about availability of computer code

Data collection	Western Blot quantification : ImageJ 1.53k.<br>Mass spectrometry data acquisition: Bruker Compass HyStar v 5.1, OToF Control Version 5.2, Chromeleon Plugin version 7.21.<br>Microscopy:

Data analysis	Statistical tests:<br>Graphpad Prism v10<br>Mass spectrometry data analysis:<br>DIA-NN version 1.8<br>ProteoWizard MS Convert GUI version 3<br>MSFragger version 3.5<br>Philosopher version 4.4.0<br>Ionquant version 1.8.0<br>Percolator version 3.05.0<br>Perseus version v1.6.15.0<br>Spectronaut v19.5.24116.62635<br>Rain Cloud Plot Shiny app (https://github.com/gabrifc/raincloud-shiny)<br>Instant Clue (v0.12.2)<br>R version 4.4.2 and R Studio version 2023.6.1.524<br>code available: https://github.com/MiguelCos/BAG3_turboID_proteomics-Differential-abundance-analysis

For manuscripts utilizing custom algorithms or software that are central to the research but not yet described in published literature, software must be made available to editors and reviewers. We strongly encourage code deposition in a community repository (e.g. GitHub). See the Nature Portfolio guidelines for submitting code & software for further information.

Data

Policy information about availability of data

All manuscripts must include a data availability statement. This statement should provide the following information, where applicable:

- Accession codes, unique identifiers, or web links for publicly available datasets
- A description of any restrictions on data availability
- For clinical datasets or third party data, please ensure that the statement adheres to our policy

The mass spectrometry proteomics data have been deposited to the ProteomeXchange Consortium (Pubmed ID: 36370099) via the PRIDE partner repository (PubMed ID: 34723319) with the the dataset identifiers PXD044976 for the human fractionated muscle proteome and phosphoproteome (Reviewer account username: reviewer_pxd044976@ebi.ac.uk; Password: W8G1qGrD) and PXD074000 for the BAG3 proximity biotinylation data (Reviewer account username: reviewer_pxd074000@ebi.ac.uk; Password: y4tzsZWlSMQq).

Research involving human participants, their data, or biological material

Policy information about studies with human participants or human data. See also policy information about sex, gender (identity/presentation), and sexual orientation and race, ethnicity and racism.

Reporting on sex and gender	All subjects identified themselves with their sex and gender written on their individual and signed form of consent. Subjects in our study identified themselves as he/him (n=7) or she/her (n=1)<br><br>Sex based differences were not relevant in this study because the investigated topic of skeletal muscle proteostasis after acute resistance exercise has to date not been described to be differentially regulated between sexes.
Reporting on race, ethnicity, or other socially relevant groupings	There was no grouping conducted concerning race, ethnicity, sex or other socially relevant factors. We used only one group during the study and the control or reference point was the resting biopsy before the first acute resistance exercise (SMO)<br><br>Due to the nature of the topic of our approach the most important confounding factor was any form of resistance exercise within 24 hours before SMO (intense resistance exercise with biopsies). To control for that issue all subjects were advised to refrain from any form of resistance exercise 4 weeks before the study and throughout the entire study except the training that was prescribed in our training regimen.
Population characteristics	The study population consisted of young healthy subjects (7male/ 1 female; 24±4,2 years; 184±3,2 cm; 81,5±7 kg see Table S1) All subjects were physically active and regularly conducting sports.<br><br>Participants were healthy, physically active volunteers recruited for an intensive resistance exercise and repeated muscle biopsy study. Therefore, some self-selection bias cannot be excluded, potentially limiting generalizability to broader populations. However, this is unlikely to substantially affect the mechanistic observations related to acute resistance exercise-induced muscle responses under the highly standardized experimental conditions.<br>Subjects received 300€ as compensation for the entire study.
Recruitment	All participants were recruited via an online advertisement platform of the website from the German Sport University Cologne
Ethics oversight	The ethics proposal was approved by the ethics commitee of the German Sport University Cologne.<br>All subjects provided written informed consent to participate after receiving oral and written explanations regarding the study's purpose and the potential risks associated with their participation.

Note that full information on the approval of the study protocol must also be provided in the manuscript.

Field-specific reporting

Please select the one below that is the best fit for your research. If you are not sure, read the appropriate sections before making your selection.

☒ Life sciences ☐ Behavioural & social sciences ☐ Ecological, evolutionary & environmental sciences

For a reference copy of the document with all sections, see nature.com/documents/nr-reporting-summary-flat.pdf

Life sciences study design

All studies must disclose on these points even when the disclosure is negative.

Sample size	We did not conduct a power analysis and instead collected a sample size of 8 subjects that is frequently used in human muscle biopsy studies. We observed in previous studies that acute resistance exercise induced changes in protein phosphorylation that could be resolved by similar cohort sizes.
Data exclusions	Dara from two subjects were excluded during mass spectrometry data analysis (one male, one female). The reason for this stratification is described in the manuscript.
Replication	For the human cohort, results obtained by mass spectrometry were validated by immunohistochemical analysis and immunoblotting of independent protein extractions from additional cryosections of the same muscle specimen. Cell biological experiments were performed 6 times with similar results.
Randomization	We used only one experimental group, randomization was therefore not possible.
Blinding	Not applicable as only one group was collected.

Reporting for specific materials, systems and methods

We require information from authors about some types of materials, experimental systems and methods used in many studies. Here, indicate whether each material, system or method listed is relevant to your study. If you are not sure if a list item applies to your research, read the appropriate section before selecting a response.

Materials & experimental systems

n/a	Involved in the study	
☐	☒	Antibodies
☐	☒	Eukaryotic cell lines
☒	☐	Palaeontology and archaeology
☒	☐	Animals and other organisms
☒	☐	Clinical data
☒	☐	Dual use research of concern
☒	☐	Plants

Methods

n/a	Involved in the study	
☒	☐	ChIP-seq
☒	☐	Flow cytometry
☒	☐	MRI-based neuroimaging

Antibodies

Antibodies used	described in Supplementary table S7
Validation	All commercial antibodies were used according to the validation statement of the manufacturers.<br>The BAG3 antibody generated by the Höhfeld lab is validated in the publication Arndt et al, Current Biol 2010 and specificity has been demonstrated by siRNA depletion in Ulbricht et al., Current Biol 2013.<br>The XIRP1 antibody was validated on human cardiac muscle extracts and is described in "Van der Ven et al., Exp. Cell. Res. 306 (2006) 2154-2167" (doi: 10.1016/j.yexcr.2006.03.015).<br>The Filamin C antibody was generated and validated by the Fürst lab. Since 2000 it was used in many publications (PMID: 11038172; PMID: 3288764)

Eukaryotic cell lines

Policy information about cell lines and Sex and Gender in Research

Cell line source(s)	C2 cells were described in Yaffe and Saxel Nature 1977, for this study obtained from Dieter Fürst´s laboratory, Bonn.

Authentication	Morphologically by microscopy and by testing for expression of muscle-specific proteins
Mycoplasma contamination	The cell line tested negative for mycoplasma contamination.
Commonly misidentified lines (See ICLAC register)	none

Plants

Seed stocks	*Report on the source of all seed stocks or other plant material used. If applicable, state the seed stock centre and catalogue number. If plant specimens were collected from the field, describe the collection location, date and sampling procedures.*
Novel plant genotypes	*Describe the methods by which all novel plant genotypes were produced. This includes those generated by transgenic approaches, gene editing, chemical/radiation-based mutagenesis and hybridization. For transgenic lines, describe the transformation method, the number of independent lines analyzed and the generation upon which experiments were performed. For gene-edited lines, describe the editor used, the endogenous sequence targeted for editing, the targeting guide RNA sequence (if applicable) and how the editor was applied.*
Authentication	*Describe any authentication procedures for each seed stock used or novel genotype generated. Describe any experiments used to assess the effect of a mutation and, where applicable, how potential secondary effects (e.g. second site T-DNA insertions, mosiacism, off-target gene editing) were examined.*
