# Supplementary material for: Fractionated proteomics identifies a protein network mitigating resistance exercise-induced damage in human skeletal muscle
Source: Nat Commun. 2026 Jul 28;17:7110. doi: 10.1038/s41467-026-75501-y (PMC13415819; doi:10.1038/s41467-026-75501-y)
Supplement: Supplementary file 10 — Transparent Peer Review file [file 41467_2026_75501_MOESM10_ESM.pdf]

nature portfolio

Peer Review File

Fractionated proteomics identifies a protein network mitigating resistance exercise-induced damage in human skeletal muscle

Corresponding Author: Professor Sebastian Gehlert

This file contains all reviewer reports in order by version, followed by all author rebuttals in order by version.

Version 1:

Reviewer comments:

Reviewer #1

(Remarks to the Author)
This manuscript presents a comprehensive and mechanistically detailed investigation into the molecular and structural responses of human skeletal muscle to resistance exercise (RE)-induced damage. By examining muscle samples in unadapted, adapted, and deadapted states, the authors provide valuable insights into the dynamic remodeling of the sarcomeric cytoskeleton and associated protein networks. Their identification of a functional complex centered on BAG3, involving key mechanosensory, chaperone, and lipid-associated proteins, significantly advances our understanding of how muscle maintains homeostasis under mechanical stress. The integration of proteomic, phosphoproteomic, and structural analyses reflects a commendable level of rigor, and the findings contribute meaningfully to the field of muscle biology. Overall, this is a good study that demonstrates an observation in human skeletal muscle that is then examined with in vitro studies using C2C12 cells. This reviewer’s main concerns are related to the human skeletal muscle data, the study design and the relevance of the in vitro model to the human findings. See comments below:

Comment 1: Given the established sex differences between male and female skeletal muscle, it would be useful if the authors justified the study design used with 7 male subjects and 1 female subject. As is shown in Figure S2D, the female subject is quite distinct from the male subjects. Why was a female subject included in the study? Why were additional female subjects not included? Could the mechanisms described in the manuscript depend on sex? If the mechanisms described do not depend on sex, what would the rationale for excluding the female subject be?

Comment 2: Two subjects have been removed from the analysis (according to Table S1), but this is not thoroughly explained in the manuscript. Unless I missed it, the only discussion of subject exclusion is in Table S1 and not in the text. This should be made more clear to the reader, and should have additional text to justify the exclusion of these subjects. Apart from the female subject clustering away from the males and one male subject having a different MHC isoform distribution, are there other reasons to exclude? Would these factors influence how the muscle is damaged? These discussion points should be added.

Comment 3: It is explained that MHC isoform content differs between one male subject and the other subjects. However, it is not discussed in the manuscript if there are different mechanisms of damage for different fiber types. Is this the case? The relevance of fiber type on downstream analyses and conclusions should be made more clear - are the findings influenced by fiber type distribution? Do the mechanisms differ between fiber types? Additionally, if MHC does influence these damage mechanisms, a characterization of the MHC isoforms in the C2C12 cells should be incorporated/discussed.

Comment 4: The rationale for examining the two different proteomes (detergent-resistant and soluble) should be made more clear. It appears that the theory is that the insoluble fraction is associated with clusters of proteins related to muscle damage, but this should be clarified in the text. Is this an established method in the literature? Do these proteins become less soluble when they associate with the cytoskeleton?

Comment 5: Is what we are observing an upregulation of these proteins in the muscle in response to damage or is this a redistribution of the proteins within the muscle (i.e. from soluble to insoluble)? Could this be made clearer in the manuscript?

Comment 6: The in vitro studies provide a valuable insight into the mechanisms behind these damage-responsive pathways. However, since the in vitro studies relied on pharmacological agents to inhibit autophagy, the direct link between mechanical stress/damage and the abundance/localization of these proteins is difficult to show. Is it possible to show this mechanical-damage mechanism in an in vitro setting (i.e. mechanically stress the cells)? Alternatively, could additional text be added that addresses this concern? Lines 467-479 seem to focus on the idea that the muscle is mechanically damaged, but this mechanical damage is not present in the in vitro model (or, perhaps this reviewer is not able to link the pharmacological intervention to mechanical damage/strain).

Minor comment: Clarification on how muscle biopsies were frozen would be helpful (line 142). The sections look nicely stained, however when biopsies are simply frozen in liquid nitrogen, the structure can be affected. Was this performed in liquid nitrogen-cooled isopentane perhaps (this is mentioned later in the methods).

Minor comment: There was no muscle hypertrophy present (as measured by myofibrillar area), but muscle strength did increase (Figure 1b). Comments on this?

Minor comment: Related to figure 1b: One subject seems to not have a large increase in torque after the training period, but then has a dramatic increase following detraining? Figure 1c also seems to present an inconsistent pattern for one of the subjects.

Reviewer #2

(Remarks to the Author)
The paper starts out with the notion that resistance exercise results in extensive remodeling of skeletal muscle in cytoskeletal, sarcomeric and stress/metabolic proteins and explores this adaptive responses after acute and repeated resistance exercise (6 weeks) as well as after 3 weeks of detraining. The study follows an unbiased design in which 7 males and 1 female are subjected to a series of controlled resistance exercises for 6 weeks without progressive overlaod. Prior to the first, after the first, after 6 weeks of training (pre and post the final exercise session) and before and after an acute exercise session after here weeks of detraining muscle biopsies were taken from the vastus lateralis muscle. Using immunoblotting, immunolocalisation and (Phospho)-Proteomics changes were monitored in an unbiased fashion in muscle fractions enriched in soluble cytoskeletal and associated proteins as well as in the pellet of these fractions.
Some of the stress-responsive proteins are involved in chaperone assisted selective autophagy and hence believed to be under control of BAG3, this was studied in more detail by a siRNA based knock-down of BAG3 in C2C12 cells. Similarly, the putative role of PDLIM3, PLIN5 and XIRP during chaperone assisted selective autophagy was studied in C2C12 cells.
The study lacks a clear hypothesis and is rather explorative in nature. The approach and the cell studies hooked up to the human experiment together do deliver an enormous data-set which opens many avenues for follow-up studies. Call me old-fashioned, but I tend to think that the wide range or processes, proteins and number of leads that have been followed in the present paper hampers to send a clear message to the reader. In my opinion, the paper would clearly benefit (and get a required improvement in readability) if the work is rewritten in a hypothesis oriented fashion with fewer data and fewer leads which are followed and discussed in more detail. I think the overload of data and pathways studied makes it almost impossible for the reader/reviewer to judge the quality and relevance of the different components. As an example I will refer to the statement on page 13 line 443-447 that 'together our physiological, proteomic, p-proteomic Immunohistocemical and cell biological data reveals a network of proteostasis factors that maintain and restore sarcomeric integrity, this pathway is centered around the chaperone assisted selective autophagy'. In fact, I think the current study was not needed to make that statement. Based upon the literature that is out, one could have made a very similar statement without doing this study.
In its current version, the paper also does not permit in depth examination of what is really going on and hence might miss-out on relevant pathways that now pop-up somewhat unexpectedly, and are 'only' explored in their conventional context, whilst not attempting to examine the putative biological role in more detail. I will use the (in my opinion) somewhat unexpected observation that PLIN5 is highly responsive to the type of exercise performed. Indeed, in skeletal muscle PLIN5 associates with LD's, can be found on LD-mito contact sites, in the cytosol (possibly bound to micro LD's and in nuclei. It can be found as a p-protein at Ser155 to regulate PKA-mediated lipolysis. From the data it is not clear in which cytosolic compartment these changes do take place, nor if changes at the phosphor proteome are detected. The next step, to knock-down the gene in C2C12 cells, is quite a leap (next to the fact that as far as I am aware of, PLIN5 is barely detectable in C2C12 cells, neither at the gene nor at the protein level). One might thus doubt if this cell model is the best to explore the putative role of PLIN5 in the processes mentioned. Given that some studies have reported PLIN5 in nuclei as well, and given the fact that upon damaging exercise, there is quite some nuclear accretion to the affected muscle sites with lots of acylated histones, one could also hypothesize a role for PLIN5 in delivery of acetyl groups to the nuclei that are accreted to the affected muscle sites and thus be part of the adaptive process. I know this is a highly speculative approach, but it might deserved in-depth exploration, whereas in the current paper the PLIN5 part is mentioned but at the same time it is sort of lost in the follow-up.
I pick out the PLIN5 part as I know a little on it and can hypothesize freely, it is likely that others that ore more familiar with the other proteins you are playing with can do the same with their pep-proteins. My concern with the current paper is the fact that a lot of data is added to the field, but with limited knowledge advance, and in fact this may prevent others from exploring the more in-depth stuff.
Long story to say I truly and deeply respect all the hard work and effort that has been put into this paper, but I am missing a more in depth analysis, which really would result in knowledge gain on one or two of that pathways explored.

Reviewer #3

(Remarks to the Author)
This is a comprehensive and technically robust study that successfully integrates human physiology, advanced proteomics, and cell biology to elucidate a novel protein network mediating skeletal muscle responses to mechanical stress. Its experimental design—particularly the "adaptation-deadaptation" model—stands out as a key strength. The findings are novel and hold significant implications for advancing our understanding of muscle homeostasis and myopathic mechanisms. However, regarding mechanistic depth and the generalizability of its conclusions, several critical aspects warrant further refinement and clarification.
1.The study effectively identifies a co-regulated network but falls short of definitively establishing direct mechanistic connections between the newly identified components (PDLIM3, XIRP1, PLIN5) and the core CASA machinery. To confirm physical interactions—for instance, between PDLIM3 and BAG3, or PLIN5 and autophagosomal markers—experiments such as co-immunoprecipitation (Co-IP) or proximity ligation assays (PLA) should be performed using human muscle lysates or mechanically stimulated myotubes.
2.The identified phosphosites (e.g., those on XIRP1 and PDLIM3) are of interest, but their functional relevance remains solely correlative. To enhance the study's impact, key phosphosites should be mutated, and the resulting effects on protein localization, protein-protein interactions, and autophagic flux should be assessed in the C2C12 myotube model.
3.All functional validation experiments were conducted exclusively in a murine myotube model. Although this model provides valuable insights, it creates a critical gap in demonstrating the functional necessity of this protein network in an in vivo mammalian system subjected to mechanical stress. Knocking down a key component (e.g., Plin5 or Pdlim3) in mouse muscle—for example, via AAV-mediated shRNA—followed by a damaging exercise regimen would yield far more robust evidence supporting the network's physiological function.
4.The human cohort size (n=8) is relatively small for a multi-timepoint omics study, and the inclusion of only one female subject (M10) represents a notable limitation. The clear outlier behavior of M10 in the principal component analysis (PCA; Fig. S2d) must be discussed, as it may reflect a potential sex-specific effect. In the Discussion section, the authors must explicitly acknowledge that the small sample size and sex imbalance limit the generalizability of the findings, and frame this as a key study limitation.
5.Kinase Substrate Enrichment Analysis (KSEA) was used to predict kinase activities. For the most compelling predictions —such as increased PRKG1 activity—follow-up experiments are needed: either validate the phosphorylation status of well-established downstream substrates of these kinases in the study samples, or use pharmacological kinase inhibitors in the cell model to determine whether they block the exercise-induced phosphorylation of the key sites identified.

Version 2:

Reviewer comments:

Reviewer #1

(Remarks to the Author)
The authors have addressed my concerns raised during the initial review, providing justifications for the study design and reinforcing the manuscript with new experimental data. Most notably, the inclusion of electrical pulse stimulation (EPS) experiments in the in vitro models effectively bridges the gap between human observations and molecular mechanisms. By clarifying the methodology behind the proteomic fractionation and transparently discussing the limitations regarding sex and fiber-type variability, the authors have significantly strengthened the rigor and clarity of the paper.

Positive: The addition of the Damage EPS cell culture data strengthens the mechanistic link between mechanical strain and the CASA pathway.

Acceptable: While the exclusion of the female participant is a limitation regarding the study's generalizability, their justification (lower lesion percentage and proteomic clustering) is scientifically sound for a mechanistic study of this size.

The manuscript is improved. The added clarity regarding protein redistribution (soluble vs. pellet) and the honest discussion of fiber-type limitations make the findings more robust.

Reviewer #3

(Remarks to the Author)
The authors have satisfactorily addressed my concerns. No further questions; I recommend acceptance.

Open Access This Peer Review File is licensed under a Creative Commons Attribution 4.0 International License, which permits use, sharing, adaptation, distribution and reproduction in any medium or format, as long as you give appropriate credit to the original author(s) and the source, provide a link to the Creative Commons license, and indicate if changes were made.
In cases where reviewers are anonymous, credit should be given to 'Anonymous Referee' and the source.
The images or other third party material in this Peer Review File are included in the article’s Creative Commons license, unless indicated otherwise in a credit line to the material. If material is not included in the article’s Creative Commons license and your intended use is not permitted by statutory regulation or exceeds the permitted use, you will need to obtain permission directly from the copyright holder.
To view a copy of this license, visit https://creativecommons.org/licenses/by/4.0/

We thank the editor and the reviewers for their detailed comments and suggestions that helped us to improve the clarity and impact of our manuscript. Point by point responses to each reviewer comments are appended below.

Reviewer #1 (Remarks to the Author):

This manuscript presents a comprehensive and mechanistically detailed investigation into the molecular and structural responses of human skeletal muscle to resistance exercise (RE)-induced damage. By examining muscle samples in unadapted, adapted, and deadapted states, the authors provide valuable insights into the dynamic remodeling of the sarcomeric cytoskeleton and associated protein networks. Their identification of a functional complex centered on BAG3, involving key mechanosensory, chaperone, and lipid-associated proteins, significantly advances our understanding of how muscle maintains homeostasis under mechanical stress. The integration of proteomic, phosphoproteomic, and structural analyses reflects a commendable level of rigor, and the findings contribute meaningfully to the field of muscle biology. Overall, this is a good study that demonstrates an observation in human skeletal muscle that is then examined with in vitro studies using C2C12 cells.

We thank the reviewer for emphasizing the comprehensiveness, novelty and importance of our study.

This reviewer's main concerns are related to the human skeletal muscle data, the study design and the relevance of the in vitro model to the human findings. See comments below:

Comment 1: Given the established sex differences between male and female skeletal muscle, it would be useful if the authors justified the study design used with 7 male subjects and 1 female subject. As is shown in Figure S2D, the female subject is quite distinct from the male subjects. Why was a female subject included in the study? Why were additional female subjects not included? Could the mechanisms described in the manuscript depend on sex? If the mechanisms described do not depend on sex, what would the rationale for excluding the female subject be?

We thank the reviewer for this important comment. To date, only limited unbiased proteomic data are available on sex-specific differences in human skeletal muscle. Although previous studies have reported differences in mitochondrial and glycolytic protein abundance between males and females (PMID: 37670389), these differences appear to be largely explained by sex-related differences in fiber type composition, with males showing a higher proportion of fast glycolytic (type II) fibers and a lower proportion of slow oxidative (type I) fibers (PMID: 40516819). However, so far there was no indication of contraction- or resistance exercise-induced differences between the male and female skeletal muscle proteomes, particularly with respect to proteins involved in sarcomere proteostasis.

From the outset, we aimed to include female participants in the study, but were not able to recruit a larger number of volunteers during the study period. To maximize sample size, we initially included all available skeletal muscle samples in the analyses. In our Western blot and immunohistochemical analyses, we did not observe obvious differences between the female participant and the male participants. In contrast, the unbiased proteomic analyses revealed a distinct clustering, and immunohistology a markedly lower extent of myofibrillar damage. In the female participant, both force generation and skeletal muscle mass were lower than in the male participants. We initially assumed that this might still result in a comparable degree of damage in the vastus lateralis. However, as shown in the source data for Fig. 1f, only 4% of fibers displayed lesions after SMO in the female participant, compared with approximately 16% in the male participants. This indicates that sex-related differences in the response to RE may exist, which will have to be addressed in a future study with a larger number of female participants.

As the sex-related differences would increase the variability of the dataset and impede identification of RE-induced proteome changes, we decided to exclude the female dataset from the present analysis. We now describe this in more detail in the main text on page 6:

"The female participant was initially included to maximize sample size, as no prior data suggested sex-specific proteomic responses under the applied conditions. However, we observed that the female participant had only 4% of fibers associated with myofibrillar lesions, which was approximately threefold lower than in the male participants. As this difference likely influenced the regulation of proteostasis factors after SMO in our study the data from the female participant were excluded from further analyses."

Comment 2: Two subjects have been removed from the analysis (according to Table S1), but this is not thoroughly explained in the manuscript. Unless I missed it, the only discussion of subject exclusion is in Table S1 and not in the text. This should be made more clear to the reader, and should have additional text to justify the exclusion of these subjects. Apart from the female subject clustering away from the males and one male subject having a different MHC isoform distribution, are there other reasons to exclude? Would these factors influence how the muscle is damaged? These discussion points should be added.

We agree that the exclusion criteria should be clear for the reader. The reason for removing the data of the female subject has been discussed above. The exclusion of the male participant with a very high proportion of fast myosin heavy chain (MHC) isoforms (approximately 70–80%) was based on our previous human training studies involving skeletal muscle biopsies, where we frequently observed an approximately balanced distribution of slow and fast fibers (~50/50%), and accumulating data in the literature.

A central aim of the present study was to investigate the response of skeletal muscle to mechanical stimulation without introducing a priori hypotheses regarding fiber type–specific responses of proteostasis factors. The repeated biopsy design, the demanding recruitment process, and ethical constraints prevented an initial fiber type screening. It was therefore not feasible to pre-select or stratify participants according to their baseline fiber type composition. Although the extent of myofibrillar lesions in the excluded subject (M1) was comparable to that observed in the other male participants (see source data for Fig. 1f), accumulating proteomic evidence indicates substantial differences between type I and type II muscle fibers (PMID: 34727990), including differences in proteins involved in proteostasis pathways (PMID: 28614723). In line with these reports, the additional variability introduced by the data of the subject with unusually high proportion of fast fibers prevented reliable detection of RE-induced changes in the proteomics dataset. Therefore, we decided to exclude this dataset from further analysis, which we now explain in greater detail on page 6:

"Fibre type-specific differences of proteostasis factors have been reported during aging and under resting conditions[28], where BAG3 and HSPB5 abundance is reduced in fast fibers compared to slow fibers. Since our sampling did not allow us to assess the impact of extreme variations in fiber type on the proteome signature in young muscle, we excluded this dataset from further analysis to minimize variability."

We further acknowledge this limitation on p7:

"We did not differentiate lesion formation between type I and II fibers and whether the mechanism of damage may differ between them. However, the fiber type distribution in the stratified cohort of six subjects was very similar, and we therefore anticipate consistent responses."

We have also added a note on page 16 in the end of the results/discussion section that a systematic analysis of sex- and fiber type–specific proteome responses will be important questions for future studies.

Comment 3: It is explained that MHC isoform content differs between one male subject and the other subjects. However, it is not discussed in the manuscript if there are different mechanisms of damage for different fiber types. Is this the case?

The relevance of fiber type on downstream analyses and conclusions should be made more clear - are the findings influenced by fiber type distribution? Do the mechanisms differ between fiber types? Additionally, if MHC does influence these damage mechanisms, a characterization of the MHC isoforms in the C2C12 cells should be incorporated/discussed.

It is indeed an important and interesting question whether the nature or magnitude of damage differs between fast- and slow-twitch fibers. The immediate cause of damage

in both fiber types most likely arises from tension-induced strain at the level of the Z-disks in all recruited muscle fibers. How mechanical tension is exerted and distributed across the recruited fiber population remains largely unknown. Schiaffino et al. (PMID: 22013216) reported that Z-line width is smaller in fast fibers than in slow fibers. In addition, fast fibers have been described as being more susceptible to damage than slow fibers (PMID: 27032709; PMID: 11181582). Furthermore, proteome studies reported that the abundance of proteostasis-related factors including CASA components differs between type I and type II fibers, particularly during aging (PMID: 28614723). Moreover, atrophy of fast fibers has been associated with a more pronounced decline in HSPB5 levels.

As discussed above, our current sampling did not permit in-depth fibre-type resolved analysis. However, selective analysis of known and newly identified CASA proteostasis network factors (HSPB1, HSPB5, PDLIM3, PLIN5, XIRP1) in the proteome data from the two excluded subjects shows increased abundance in the pellet fraction after RE. This is consistent with protein abundance patterns observed in the stratified cohort, indicating that the involvement of these proteins in the molecular response to RE is independent of sex and fibre type (Figure R1 for review).

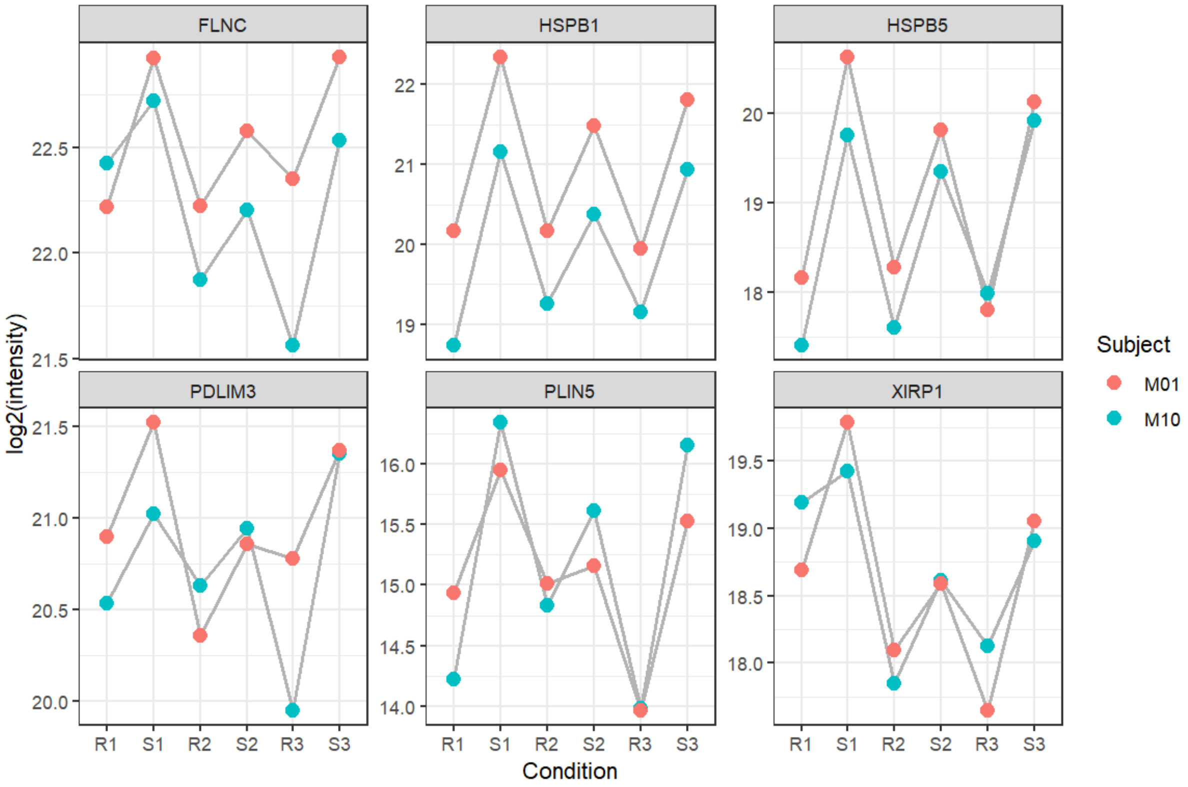

Figure R1. Protein abundance of the CASA substrate FLNC and previously established (HSPB1, HSPB5) and new (PDLIM3, PLIN5, XIRP1) CASA network members in the pellet fraction of the excluded subjects as estimated by LFQ intensity in the proteomics data.

We now discuss these limitations of our study and the requirement for a future and mechanistic fiber type specific analysis of proteostatic responses in skeletal muscle in our concluding discussion on p18:

“The human data are observational in nature and limited by cohort size; thus, we cannot exclude potential modulation by fibre type composition, sex, age or other diversity dimensions. Future studies combining fiber type–resolved proteomics or imaging approaches with resistance exercise paradigms will determine whether proteostatic responses differ systematically between fast and slow muscle fibers and sexes.“

Comment 4: The rationale for examining the two different proteomes (detergent-resistant and soluble) should be made more clear. It appears that the theory is that the insoluble fraction is associated with clusters of proteins related to muscle damage, but this should be clarified in the text. Is this an established method in the literature? Do these proteins become less soluble when they associate with the cytoskeleton?

We thank the reviewer for this suggestion. The fractionation protocol has been established by Zucker and Masiello in 1983 (PMID: 6831034). We have previously applied this protocol to show that HSPB5 is more tightly associated to muscle fibers after exposure to mechanical stress (PMID: 30920888). This condition reversed when skeletal muscle adapted after repeated RE. This indicated that damage-associated proteostasis factors indeed become less soluble by binding to the cytoskeleton and led us to apply combine this fractionation procedure with an unbiased proteome analysis.

The reference to Zucker and Masiello is included in the first paragraph on p6 and the resulting expected enrichment in cytoskeletal proteins is now emphasized:

*“To investigate RE-induced changes in protein abundance and phosphorylation state in the context of their subcellular localization, we first homogenized cryosectioned muscle samples in a buffer containing Triton X-100 and fractionated the sample by centrifugation (PMID: 6831034). As expected, this fractionation yields a soluble supernatant proteome fraction and a detergent-resistant pellet fraction enriched in cytoskeleton-associated proteins (**Fig. 1g**).”*

In addition, we describe our hypothesis more clearly in the introduction on p4:

Comment 5: Is what we are observing an upregulation of these proteins in the muscle in response to damage or is this a redistribution of the proteins within the muscle (i.e. from soluble to insoluble)? Could this be made clearer in the manuscript?

The short time to sampling after RE (within 60 min) does not allow for major protein synthesis. We therefore assumed that most changes in proteins abundance will be caused rapid change in translocation between both fractions, and that this would allow us to identify new proteostasis factors recruited to the stressed or damaged

cytoskeleton. We now better explain the rationale for that on page 4, in the second-last paragraph of the introduction.

"We therefore reasoned that identification of additional proteins with similar behaviour, i.e., force-dependent phosphorylation and increased binding to the sarcomeric cytoskeleton, could reveal new components contributing to muscle proteostasis. Because acute RE-induced mechanical stress likely alters the binding of chaperones and CASA components towards damaged or unfolded proteins of the sarcomere, we assumed their rapid redistribution towards the sarcomeric cytoskeleton after each RE bout. Consequently, we fractionated muscle samples into a soluble and sarcoplasmic/cytoskeletal fraction and analyzed RE-dependent changes in localization of those proteins as well as the proteome and phosphoproteome in both fractions."

Indeed, we observed such relocation for several proteins (see Fig. 2 b-h), and this very likely reflects of a changed binding/association behavior required for chaperone-assisted stabilization, turnover and repair of the cytoskeleton.

Comment 6: The in vitro studies provide a valuable insight into the mechanisms behind these damage-responsive pathways. However, since the in vitro studies relied on pharmacological agents to inhibit autophagy, the direct link between mechanical stress/damage and the abundance/localization of these proteins is difficult to show. Is it possible to show this mechanical-damage mechanism in an in vitro setting (i.e. mechanically stress the cells)? Alternatively, could additional text be added that addresses this concern?

Lines 467-479 seem to focus on the idea that the muscle is mechanically damaged, but this mechanical damage is not present in the in vitro model (or, perhaps this reviewer is not able to link the pharmacological intervention to mechanical damage/strain).

We are glad that the reviewer sees the value of the cell culture experiments for obtaining insights into underlying molecular mechanisms. We would like to point out that the pharmacological agents are not meant to induce any kind of stress. Instead, the addition of inhibitors of autophagic degradation represents a well-established procedure to determine autophagic turnover rates. It allowed us to determine that CASA is active in differentiated myotubes based on force generation through spontaneous contraction. We rephrased the corresponding sentences in the manuscript to clarify this aspect.

Nevertheless, we followed the suggestion of the reviewer and applied lesion-inducing stress conditions to isolated myotubes through electrical pulse stimulation (damage EPS). Orfanos et al. previously showed that damage EPS causes the formation of XIRP and FLNC positive sarcomeric lesions in C2C12 myotubes thus mirroring consequences of resistance exercise in mice and humans (PMID: 26804343). Strikingly, under damage EPS, autophagic turnover of BAG3, HSPB8, HSPB1, HSPB5 and PDLIM3 was no longer detectable (Fig. S9 a-c). The data illustrate an intensive

regulation of the CASA machinery under acute mechanical stress. The observed attenuation of CASA activity explains the accumulation of CASA clients and CASA components in sarcomeric lesions in response to resistance exercise and indicates that under acute stress rapture prevention is favored over the degradation of functionally impaired cytoskeleton proteins. We are the first to report on this acute regulation of the CASA machinery, which explains many findings obtained in observational studies on RE-responses. A corresponding chapter explaining these findings has been added to the main text.

Under damage EPS, a residual degradation activity is only observed for XIRP2 and XIRP1. The findings further emphasize the critical role of the XIRP proteins, which were previously described as damage markers, for the regulation of autophagic activity in mechanically strained muscle. This is reported in our manuscript for the first time.

Minor comment: Clarification on how muscle biopsies were frozen would be helpful (line 142). The sections look nicely stained, however when biopsies are simply frozen in liquid nitrogen, the structure can be affected. Was this performed in liquid nitrogen-cooled isopentane perhaps (this is mentioned later in the methods).

Muscle samples were frozen in liquid nitrogen precooled isopentane in a small glass beaker to get a thorough and rapid cooling of the isopentane and the muscle sample. We added this information to the text at page 5 in the last paragraph.

Minor comment: There was no muscle hypertrophy present (as measured by myofibrillar area), but muscle strength did increase (Figure 1b). Comments on this?

This is a common observation in exercise science. Increases in muscle strength occur often before any hypertrophy. The central nervous system learns to recruit more fibers and releases higher innervation frequencies to the motoric endplate. These are the major mechanisms resulting in strength gains. Concerning human RE-induced hypertrophy, Damas and colleagues have shown (PMID: 27219125) that hypertrophy is only detectable when the damage is reduced and when the subjects train for at least ten weeks (when training only twice per week). Hypertrophy is dependent on the amount of training units in a time course of several weeks. RE training, solely two times per week with high volumes of eccentric contractions as in our study, is intended to result in a provoked damage within sarcomeres without the potential to hypertrophy in six weeks. The accumulating effect of protein synthesis leading to substantial hypertrophy was likely too low and therefore not detectable.

Minor comment: Related to figure 1b: One subject seems to not have a large increase in torque after the training period, but then has a dramatic increase following detraining? Figure 1c also seems to present an inconsistent pattern for one of the subjects.

This is a very detailed and attentive observation. This phenomenon can be attributed to a delay of neuromuscular adaptations in the peripheral nervous system (PMID: 16464122). Sometimes repeated training, also twice per week, can result in an accumulating fatigue during the training period. As a result, the force measured at

baseline before the last training session of the adaptive period may not show the full-strength gains which may be detectable after full recovery. Therefore, in our case, after interruption of the training period strength gains may have showed a delayed peak. This phenomenon is used in elite sports to specifically reduce training in the last two weeks before competition in order to fully recover and get a peak in performance.

In short time frames of around 10 days of detraining, hypertrophy, when previously exerted (PMID: 35628242), will also be unchanged or slightly increase. This can be different between subjects. However, Z-disk proteins like desmin start to decrease (PMID: 32520607). Therefore, muscle strength and sarcomeric composition, which influences its resilience towards mechanical strain do not timely correspond after detraining. This explains why lesions start to rise again after detraining as detected in our study.

Reviewer #2 (Remarks to the Author):

The paper starts out with the notion that resistance exercise results in extensive remodeling of skeletal muscle in cytoskeletal, sarcomeric and stress/metabolic proteins and explores this adaptive responses after acute and repeated resistance exercise (6 weeks) as well as after 3 weeks of detraining. The study follows an unbiased design in which 7 males and 1 female are subjected to a series of controlled resistance exercises for 6 weeks without progressive overlaod. Prior to the first, after the first, after 6 weeks of training (pre and post the final exercise session) and before and after an acute exercise session after here weeks of detraining muscle biopsies were taken from the vastus lateralis muscle. Using immunoblotting, immunolocalisation and (Phospho)-Proteomics changes were monitored in an unbiased fashion in muscle fractions enriched in soluble cytoskeletal and associated proteins as well as in the pellet of these fractions.

Some of the stress-responsive proteins are involved in chaperone assisted selective autophagy and hence believed to be under control of BAG3, this was studied in more detail by a siRNA based knock-down of BAG3 in C2C12 cells. Similarly, the putative role of PDLIM3, PLIN5 and XIRP during chaperone assisted selective autophagy was studied in C2C12 cells.

The study lacks a clear hypothesis and is rather explorative in nature. The approach and the cell studies hooked up to the human experiment together do deliver an enormous data-set which opens many avenues for follow-up studies. Call me old-fashioned, but I tend to think that the wide range or processes, proteins and number of leads that have been followed in the present paper hampers to send a clear message to the reader.

In my opinion, the paper would clearly benefit (and get a required improvement in readability) if the work is rewritten in a hypothesis oriented fashion with fewer data and fewer leads which are followed and discussed in more detail. I think the overload of

data and pathways studied makes it almost impossible for the reader/reviewer to judge the quality and relevance of the different components.

We thank the reviewer for these comments as they illustrate the need to better explain the working hypothesis that motivated us to conduct this study. Our study is based on extensive previous work, where we showed that the small heat shock protein HSPB5 is highly responsive to RE, resulting in increased phosphorylation and cytoskeleton binding after RE. We therefore hypothesized that other proteins might show a similar behavior and the identification and characterization of these proteins may provide significant new insights into adaptation and deadaptation mechanisms in human muscle. This working hypothesis was fully validated in the current manuscript. The manuscript was edited extensively to emphasize this.

It was instrumental for our study to identify RE-responsive proteins in human muscle in a completely unbiased proteomics approach. Of course, this gives rise to an enormous data set. However, only a handful of proteins was shown to be consistently regulated during adaptation and deadaptation, involving changes in cytoskeleton association and phosphorylation status – as previously observed for HSPB5. Because HSPB5 was previously shown to associate with BAG3, the main component of the CASA pathway, we further hypothesized that RE-responsive proteins might participate in CASA. Strikingly, our functional studies indeed reveal that all of the identified proteins are part of the CASA proteostasis machinery. Thus, our research was hypothesis driven from the start and we now emphasize this throughout the text.

As an example I will refer to the statement on page 13 line 443-447 that 'together our physiological, proteomic, p-proteomic Immunohistocemical and cell biological data reveals a network of proteostasis factors that maintain and restore sarcomeric integrity, this pathway is centered around the chaperone assisted selective autophagy'. In fact, I think the current study was not needed to make that statement. Based upon the literature that is out, one could have made a very similar statement without doing this study.

With all respect, we completely disagree here. Our unbiased proteomic approach that was followed by an in-depth functional characterization of newly identified RE-responsive proteins, shows for the first time that the CASA proteostasis system reacts most strongly to adaptation and deadaptation in human muscle. This could not be concluded from previously published work. Moreover, with PDLIM3, XIRP1, XIRP2 and PLIN5 we identify several new CASA components, that are essential for the disposal of cytoskeleton proteins through selective autophagy. None of these components was previously linked to CASA-mediated muscle homeostasis.

In its current version, the paper also does not permit in depth examination of what is really going on and hence might miss-out on relevant pathways that now pop-up somewhat unexpectedly, and are 'only' explored in their conventional context, whilst not attempting to examine the putative biological role in more detail. I will use the (in my opinion) somewhat unexpected observation that PLIN5 is highly responsive to the type of exercise performed. Indeed, in skeletal muscle PLIN5 associates with LD's, can

be found on LD-mito contact sites, in the cytosol (possibly bound to micro LD's and in nuclei. It can be found as a p-protein at Ser155 to regulate PKA-mediated lipolysis.

From the data it is not clear in which cytosolic compartment these changes do take place, nor if changes at the phosphor proteome are detected.

The next step, to knock-down the gene in C2C12 cells, is quite a leap (next to the fact that as far as I am aware of, PLIN5 is barely detectable in C2C12 cells, neither at the gene nor at the protein level). One might thus doubt if this cell model is the best to explore the putative role of PLIN5 in the processes mentioned. Given that some studies have reported PLIN5 in nuclei as well, and given the fact that upon damaging exercise, there is quite some nuclear accretion to the affected muscle sites with lots of acylated histones, one could also hypothesize a role for PLIN5 in delivery of acetyl groups to the nuclei that are accreted to the affected muscle sites and thus be part of the adaptive process.

I know this is a highly speculative approach, but it might deserved in-depth exploration, whereas in the current paper the PLIN5 part is mentioned but at the same time it is sort of lost in the follow-up.

I pick out the PLIN5 part as I know a little on it and can hypothesize freely, it is likely that others that ore more familiar with the other proteins you are playing with can do the same with their pep-proteins. My concern with the current paper is the fact that a lot of data is added to the field, but with limited knowledge advance, and in fact this may prevent others from exploring the more in-depth stuff.

Long story to say I truly and deeply respect all the hard work and effort that has been put into this paper, but I am missing a more in depth analysis, which really would result in knowledge gain on one or two of that pathways explored.

We agree with the reviewer that each of the newly identified CASA components would require further analysis to establish its exact role in CASA-mediated protein homeostasis. We therefore significantly extended our initial manuscript in this regard (new Fig. 6 and Fig. S9). However, we did not further investigate PLIN5, as our data suggest that it is not part of the CASA core complex: i) it is not degraded by CASA – in contrast to core components such as PDLIM3 and XIRP1/2 (Figs. 4, S8 c-d), and ii) it is not detected in the vicinity of BAG3 through proximity biotinylation – in contrast to PDLIM3 and XIRP1/2 (Fig. 6 a-c). Furthermore, our manuscript shows for the first time the essential role of mechanoresponsive cytoskeleton interactors, i,e, PDLIM3 and XIRPs, for the initiation of CASA at strained sarcomeric structures. Therefore, we sought to provide additional evidence for this novel concept through a further functional characterization of PDLIM3.

In the new Figs. 6 d-f and S9 d-e, we provide evidence for a direct interaction between PDLIM3 and BAG3 and demonstrate that PDLIM3 stimulates CASA-dependent ubiquitylation. The data reveal the molecular basis for the recruitment function of PDLIM3 and its ability to stimulate CASA-mediated protein degradation.

Reviewer #3 (Remarks to the Author):

This is a comprehensive and technically robust study that successfully integrates human physiology, advanced proteomics, and cell biology to elucidate a novel protein network mediating skeletal muscle responses to mechanical stress. Its experimental design—particularly the "adaptation-deadaptation" model—stands out as a key strength. The findings are novel and hold significant implications for advancing our understanding of muscle homeostasis and myopathic mechanisms.

We thank the reviewer for this positive evaluation of our study.

However, regarding mechanistic depth and the generalizability of its conclusions, several critical aspects warrant further refinement and clarification.

1.The study effectively identifies a co-regulated network but falls short of definitively establishing direct mechanistic connections between the newly identified components (PDLIM3, XIRP1, PLIN5) and the core CASA machinery. To confirm physical interactions—for instance, between PDLIM3 and BAG3, or PLIN5 and autophagosomal markers—experiments such as co-immunoprecipitation (Co-IP) or proximity ligation assays (PLA) should be performed using human muscle lysates or mechanically stimulated myotubes.

Thank you for this suggestion. We performed proximity biotinylation using stably transfected C2C12 myotubes that expressed a TurboID-biotin ligase-BAG3 fusion protein (Fig. 6 a-c). In this unbiased proteomic approach, the RE-responsive proteins and newly-identified CASA components PDLIM3, XIRP1 and XIRP2 were detected as high scoring BAG3-associated proteins. The experiment provides an additional and independent line of evidence for the close cooperation of BAG3 with the mechanoresponsive cytoskeleton interactors. Moreover, evidence for a direct interaction between PDLIM3 and BAG3 is provided in Fig. S9 d,e, and in-vitro experiments reveal a stimulating activity of PDLIM3 in CASA-mediated protein ubiquitylation. The additional data further establish a direct mechanistic connection between BAG3 and PDLIM3.

2.The identified phosphosites (e.g., those on XIRP1 and PDLIM3) are of interest, but their functional relevance remains solely correlative. To enhance the study’s impact, key phosphosites should be mutated, and the resulting effects on protein localization, protein-protein interactions, and autophagic flux should be assessed in the C2C12 myotube model.

We agree with the reviewer that the identification of RE-regulated phosphosites in proteins essential for muscle homeostasis is very exciting and their functional relevance needs to be further explored. However, we would like to point out that phosphosite analysis was performed here to define the RE-responsive proteome and to narrow down the proteins which are most highly and most consistently regulated through adaptation and deadaptation in human muscle. This was instrumental for defining the set of proteins that was subjected to functional analysis, which eventually

led to the identification of several new CASA components. We feel that an in-depth analysis of the identified phosphosites would go far beyond the scope of our current manuscript.

3.All functional validation experiments were conducted exclusively in a murine myotube model. Although this model provides valuable insights, it creates a critical gap in demonstrating the functional necessity of this protein network in an in vivo mammalian system subjected to mechanical stress. Knocking down a key component (e.g., Plin5 or Pdlim3) in mouse muscle—for example, via AAV-mediated shRNA—followed by a damaging exercise regimen would yield far more robust evidence supporting the network's physiological function.

Knocking down experiments have been previously performed for PDLIM3 (also known as ALP; PMID: 11329061) and XIRP proteins (PMID: 17766470). Importantly, the results of these studies are fully consistent with the cellular activities assigned here to these proteins. In both cases, knock down leads to cardiomyopathy, and it was shown for ALP/PDLIM3 that it has an essential role in the embryonic development of the right ventricular heart chamber during its exposure to high biomechanical workloads in utero (PMID: 11329061). These data fully support a critical physiological role of the novel CASA components in muscle maintenance. This work is now mentioned in our manuscript.

4.The human cohort size (n=8) is relatively small for a multi-timepoint omics study, and the inclusion of only one female subject (M10) represents a notable limitation. The clear outlier behavior of M10 in the principal component analysis (PCA; Fig. S2d) must be discussed, as it may reflect a potential sex-specific effect. In the Discussion section, the authors must explicitly acknowledge that the small sample size and sex imbalance limit the generalizability of the findings, and frame this as a key study limitation.

We completely agree that the exclusion criteria should be clear to the reader, which we now explain in greater detail in the manuscript (please see answers to the comments of reviewer #1 for details). We acknowledge that the sample size is small. However, such sample sizes are actually the upper limit of what is possible in humans and under repeated biopsy sampling. Also relevant studies use often smaller or a similar sample size for their studies (PMID: 26437602, PMID: 35882232; PMID: 28614723). An extension of the sample size would certainly increase the sensitivity to detect changes between the studied conditions as well as sex- and fibre-type specific differences, but would likely not change the general findings of our manuscript. This is supported by selective analysis of the identified new CASA proteostasis network factors (HSPB1, HSPB5, PDLIM3, PLIN5, XIRP1) in the two excluded subjects, which showed increased abundance in the pellet fraction after RE, consistent with protein abundance patterns observed in the stratified cohort (see Figure R1 for review above).

5.Kinase Substrate Enrichment Analysis (KSEA) was used to predict kinase activities. For the most compelling predictions—such as increased PRKG1 activity—follow-up experiments are needed: either validate the phosphorylation status of well-established downstream substrates of these kinases in the study samples, or use pharmacological kinase inhibitors in the cell model to determine whether they block the exercise-induced phosphorylation of the key sites identified.

We completely agree with the reviewer that the phosphoproteomic data provides many intriguing leads for further studies. However, as argued above, the primary aim of our phosphosite analysis was to identify the RE-responsive proteome consistently regulated through adaptation and deadaptation in human muscle. This was instrumental for defining the set of proteins that was subjected to functional analysis, which led to the identification of several new CASA components. We feel that an in-depth analysis of specific kinases would distract from this core message and go beyond the scope of our current manuscript.
